# Small Animal Veterinarians’ Perceptions and Practices in Dog Aggression Prevention in Italy: A National Survey

**DOI:** 10.3390/ani16111739

**Published:** 2026-06-05

**Authors:** Fabrizio Iarussi, Valeria Milelli, Filippo Di Gesù, Antonella Colella, Alessandra Recchia, Serenella D’Ingeo

**Affiliations:** 1 Section of Veterinary Clinics and Animal Production, Department of Precision and Regenerative Medicine and Ionian Area, University of Bari Aldo Moro, 70010 Bari, Italy; antonella.colella@uniba.it (A.C.); alessandra.recchia1@uniba.it (A.R.); 2 Independent Researcher, 70121 Bari, Italy; valeriamilelli@gmail.com; 3 Independent Researcher, 73100 Lecce, Italy; f.digesu1@gmail.com; 4 Animal Physiology and Behavior Unit, Department of Veterinary Medicine, University of Bari Aldo Moro, 70100 Bari, Italy; serenella.dingeo@uniba.it

**Keywords:** dog aggression, public health, veterinarians, prevention, behavioral medicine, risk assessment, Italy

## Abstract

Dog aggression toward humans represents a relevant public health issue. In Italy, small animal veterinarians are expected to contribute to the early identification of at-risk situations and to aggression prevention. This study aimed to assess, through a nationwide survey, veterinarians’ perception of this role, regulatory awareness, and clinical practices related to dog aggression prevention in Italy. A cross-sectional study was conducted between November 2025 and February 2026 using an anonymous online questionnaire disseminated through provincial veterinary boards to private veterinarians working in small animal practice. A total of 1088 valid responses were analyzed using descriptive, inferential, and multivariable analyses. Almost all respondents reported frequent encounters with aggressive behavior, with more than 60% managing at least five aggressive dogs per year. Although 80% recognized an active preventive role for veterinarians, only 55% were aware of the current Ministerial Ordinance and 19% had ever submitted a report to public veterinary services. Specific training in behavioral medicine was independently associated with greater regulatory awareness and reporting behavior. Strong support also emerged for mandatory owner training and early behavioral assessment. A substantial gap persists between the preventive role assigned to veterinarians and the practical implementation of preventive tools. These findings support the need for strengthened behavioral medicine training and improved integration between private practitioners and public veterinary services.

## Simple Summary

Dog aggression toward people is a relevant public health concern, especially when severe injuries occur in children, older adults, or within the home. Veterinarians can play an important role in prevention because they often encounter dogs showing aggressive behavior before more serious incidents occur. This study surveyed more than one thousand Italian veterinarians working with companion animals to understand how they perceive this preventive role and how they manage dogs that may represent a risk. Most veterinarians reported frequent contact with dogs showing aggressive behavior and believed that their profession should contribute to prevention. However, the study also found important gaps: not all veterinarians were familiar with the current rules, and only a minority had ever reported high-risk cases to public veterinary services. Veterinarians with specific training in animal behavior were more aware of the rules and more likely to use preventive tools. These findings suggest that better training, clearer procedures, and closer collaboration between private veterinarians and public veterinary services could help identify risky situations earlier and improve the prevention of dog aggression.

## 1. Introduction

Dog attacks against humans represent a significant public health issue, with health, social, and regulatory implications. In the United States, approximately 4.5 million people are bitten each year, with around 885,000 requiring medical attention and about 20 fatalities [1]. In Italy, between 2009 and 2025, 54 deaths resulting from dog attacks were recorded [2]. Although fatal events represent the most extreme outcome within a continuum of manifestations of varying severity, they provide a useful indicator for describing the most severe dynamics of the phenomenon.

Dogs are the most common companion animals in Italian households [3]. The owned dog population is estimated at approximately 9 million individuals [4], an increase compared to the roughly 6.9 million estimated ten years earlier [5]. The expansion of the dog population and the increasing integration of dogs into the family unit have made human–animal interactions more frequent and complex, potentially affecting the risk of aggression.

The expression “dog aggression” encompasses a heterogeneous set of events. A bite is defined as any disruption of skin integrity caused by an animal’s teeth [6], whereas severe attacks are characterized by repeated biting or violent shaking of the victim, greater difficulty in stopping the attack, and an increased risk of serious injury [7]. A fatal event is defined by its outcome, namely death resulting from trauma or secondary infectious complications [2]. Dog aggression represents a complex and multifactorial behavioral phenomenon influenced by dog-related, human-related, environmental, and social factors [8,9]. Aggressive behavior may arise in different motivational contexts, including fear-related, territorial, possessive, defensive, or pain-related situations, and should therefore not be interpreted as a single or uniform condition [8]. Although aggressive episodes may result in bites and severe injuries, aggression itself constitutes part of the normal canine behavioral repertoire and may reflect adaptive responses to perceived threats or stressful situations [9]. Consequently, the early recognition of behavioral warning signs and the appropriate interpretation of aggressive responses are important components of preventive veterinary practice. Italian data indicate that fatal dog attacks disproportionately affect vulnerable groups. In the period 2009–2025, children aged 0–4 years accounted for 12 of the 54 victims (22.2%), consistent with international evidence documenting a substantial proportion of pediatric fatalities in early childhood [2,10,11]. Individuals aged over 64 years account for 23 of 54 victims (42.6%), reflecting increased vulnerability associated with comorbidities and reduced defensive capacity [1,2].

Fatal episodes occurred predominantly in private settings: 36 out of 54 cases (66.7%) took place in domestic environments, whereas 18 out of 54 (33.3%) occurred in public spaces, mainly in rural areas [2]. The involvement of stray dogs is limited (4 out of 54; 7.4%), while the vast majority of events involve owned dogs (50 out of 54; 92.6%) [2].

Unlike fatalities, for which international comparability is facilitated by the use of the ICD-10 classification (code W54) [12], Italy does not have a standardized national system for collecting data on non-fatal dog aggression incidents [2,13]. This gap limits the definition of stable and comparable epidemiological indicators and hinders the planning of prevention strategies based on integrated data.

From a regulatory perspective, the Italian approach has progressively moved away from the “breed-specific legislation” model introduced in the early 2000s [14,15], toward a system centered on owner responsibility and individual dog assessment [16,17,18]. Within this framework, owners may bear civil and criminal liability for damage caused by the animal [19,20,21,22,23], while public veterinary services are required to activate assessment and risk management pathways in cases of biting or aggression [16]. The current Ordinance assigns veterinarians a role in informing owners and reporting dogs requiring behavioral assessment to ASL Veterinary Services [17,18]. However, standardized operational procedures and a structured national data collection system are currently lacking. Although veterinarians are expected to play a role in primary prevention and in the early identification of at-risk situations, little is known about how this role is implemented in clinical practice. There is no systematic evidence regarding the level of awareness of the current regulatory framework, the perception of the preventive role, or the actual use of available operational tools, including reporting to Veterinary Services.

Despite the increasing public health relevance of severe dog aggression episodes in Italy and the progressive evolution of the national regulatory framework [2,14,15,16,17,18], limited evidence is currently available regarding how veterinarians perceive and implement their preventive role in clinical practice. To the authors’ knowledge, no previous nationwide study has specifically investigated the perspectives, regulatory awareness, and operational practices of Italian small animal veterinarians regarding dog aggression prevention. Exploring these aspects may provide useful insights into potential critical issues within the current prevention system and contribute to the development of more consistent preventive and educational strategies in both clinical and public health contexts.

The present study aimed to investigate, through a questionnaire administered to small animal veterinarians working in Italy: (i) the perception of the veterinary role in the prevention of dog aggression; (ii) awareness of the current regulatory framework; and (iii) the operational practices adopted in the management of dogs exhibiting aggressive behavior. The study also aimed to identify potential critical issues and areas for improvement within the current national prevention framework and to explore whether regulatory awareness and preventive practices differed according to professional characteristics and specific training in behavioral medicine.

## 2. Materials and Methods

### 2.1. Study Design

A cross-sectional observational study was conducted through the administration of an anonymous online questionnaire (Computer-Assisted Web Interviewing, CAWI), targeting small animal veterinarians working in Italy. Data were collected between November 2025 and February 2026. The target population consisted of veterinarians registered with the Italian professional boards and practicing privately in small animal medicine. According to data from the National Federation of Italian Veterinary Orders (Federazione Nazionale degli Ordini dei Veterinari Italiani, FNOVI), there are 35,669 registered veterinarians; 73% are engaged in private practice, and among these, 83% work primarily in small animal practice. Based on these data, the estimated target population is approximately 21,600 professionals [24].

Participants were recruited through institutional channels by sending a request for collaboration to the provincial veterinary boards, which forwarded to their members a communication including a description of the study and a link to access the questionnaire. Because dissemination was performed independently by the provincial veterinary boards, the exact number of veterinarians who actually received the invitation could not be determined. Therefore, a formal response rate could not be calculated.

Participation was voluntary and anonymous, with no incentives provided. Access to the questionnaire was allowed exclusively through the link distributed by the professional boards to ensure that participants belonged to the target population. Data were collected anonymously in compliance with the applicable regulations on personal data protection.

### 2.2. Ethical Considerations

The questionnaire did not involve the collection of identifiable or sensitive data. Prior to completion, participants received an information notice describing the aims of the study, and their consent was required for the use of the data for scientific research purposes. Participants who did not provide consent were unable to proceed with the questionnaire, and no responses were recorded.

### 2.3. Sample Size

The minimum sample size was calculated based on an estimated population of 21,600 individuals. Using the standard formula for sample surveys, with an expected proportion of 0.5, a confidence level of 95% (Z = 1.96), and a margin of error of 5%, and applying the finite population correction, the minimum required number of responses was 378.

Scenarios with lower margins of error were also considered: 584 responses for a 4% margin, 1017 for 3%, 2161 for 2%, and 6648 for 1%.

### 2.4. Screening and Analytical Sample

A total of 1229 questionnaires were collected. An eligibility screening step was integrated into the questionnaire structure. After the informed consent section, participants were asked whether they were professionally engaged in small animal practice. Only respondents answering “yes” were allowed to proceed with the questionnaire, whereas negative responses automatically terminated the survey.

This screening procedure was necessary because the invitation was disseminated through the provincial veterinary boards to all registered veterinarians, including professionals working in other sectors such as livestock production, public veterinary services, academia, or industry. Consequently, only veterinarians declaring professional activity primarily related to companion animals were considered eligible for inclusion in the analytical sample.

**Figure 1:**
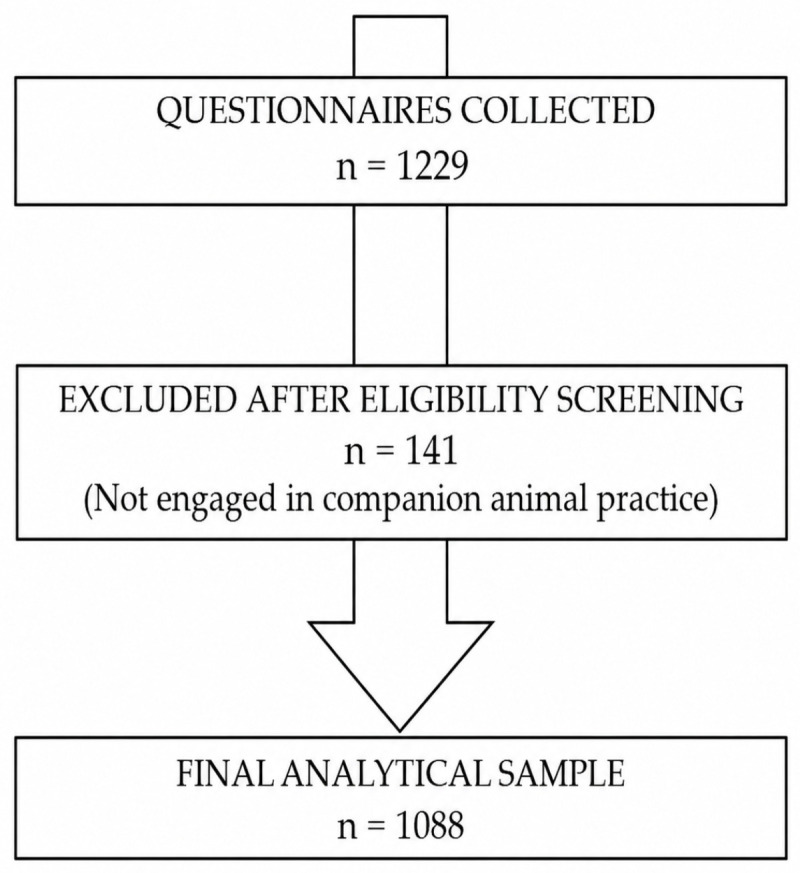
Flow diagram of participant selection and eligibility screening.

Of the 1229 responses, 1088 (88.5%) were deemed eligible, while 141 (11.5%) were excluded. The final analytical sample therefore consisted of 1088 veterinarians (Figure 1).

Considering the target population, this sample size allows estimates with high statistical precision, with a margin of error close to 3%.

### 2.5. Questionnaire Structure

The questionnaire was structured into four sections: professional characteristics (geographical area, professional role, and specific training in behavioral medicine); clinical experience with aggressive dogs (frequency of cases, depth of behavioral history taking in the domestic context, and attention to the presence of vulnerable individuals); regulatory and decision-making aspects (awareness of the current regulatory framework, perception of the veterinarian’s preventive role, management of at-risk cases, and the possibility of reporting to the Veterinary Services of the Local Health Authority (Azienda Sanitaria Locale, ASL); and risk perception and preventive tools (opinions on the introduction of mandatory training programs for owners and structured behavioral assessments). Questions were predominantly closed-ended (dichotomous or multiple-choice). A final open-ended section was also included to collect observations and operational suggestions.

The questionnaire was developed by the authors based on the objectives of the study, the current Italian regulatory framework regarding dog aggression prevention, and relevant literature in veterinary behavioral medicine.

Before dissemination, the questionnaire underwent internal review within the research team to assess the clarity, consistency, and relevance of the items. As the survey was exploratory in nature and primarily descriptive, and did not include psychometric scales or latent constructs, a formal validation process was not performed.

The questionnaire was originally developed and administered in Italian. The complete questionnaire is provided as Supplementary File S1 and is also accessible through the link reported in the Data Availability Statement section.

### 2.6. Statistical Analysis

Variables were analyzed using descriptive statistics, with absolute frequencies and percentages used to summarize categorical variables.

Associations between categorical variables were assessed using the chi-square (χ^2^) test. Statistical significance was set at *p* < 0.05. For associations found to be significant, effect size was calculated using Cramer’s V coefficient in order to quantify the strength of the relationship between variables. Additionally, two multivariable binary logistic regression models were performed to evaluate predictors of regulatory awareness and reporting behavior. The first model assessed predictors of awareness of the Ministerial Ordinance of 6 August 2013 (aware vs. not aware). The second model assessed predictors of reporting behavior, defined as having submitted at least one report to ASL Veterinary Services versus no report. Respondents indicating that reporting was “not their responsibility” were included in the no-report category. Independent variables included specific training in behavioral medicine, geographic macroarea, professional role, and frequency of clinical encounters with dogs showing aggressive behavior. This latter variable reflected the frequency with which veterinarians encountered dogs displaying aggressive behavior during routine clinical activity, regardless of whether the consultation was primarily behavioral in nature. Responses were categorized as fewer than 5, 5–10, and more than 10 encounters in the previous year. “Do not recall” responses were treated as missing values and excluded from regression analyses. Awareness of the Ministerial Ordinance was additionally included as an independent variable in the reporting behavior model. Results were expressed as odds ratios (ORs), 95% confidence intervals (CIs), and *p*-values.

Bivariable inferential analyses were additionally performed to assess the association between specific training in behavioral medicine and awareness of the current regulatory framework, between regulatory awareness and awareness of reporting procedures to ASL Veterinary Services, as well as between behavioral training and professional behavior, defined as having made at least one report. These analyses were limited to the variables considered most relevant for evaluating the relationships between training, regulatory awareness, and professional behavior. Detailed descriptive and inferential results are presented in the Supplementary Materials (Supplementary File S2, Figures S1–S25).

## 3. Results

The analytical sample consisted of 1088 veterinarians working in small animal practice.

### 3.1. Geographic Distribution and Professional Profile (Questions 1–4)

Questions 1–4 explored the geographical distribution of the sample, professional role, and the presence of specific training in behavioral medicine.

#### 3.1.1. Regional and Provincial Distribution

The regional distribution shows broad territorial coverage. Piedmont is the most represented region (219 responses; 20.1%), followed by Lombardy (165; 15.2%) and Tuscany (98; 9.0%). The least represented regions are Aosta Valley (4; 0.4%), Basilicata (5; 0.5%), and Molise (7; 0.6%). All Italian regions are represented in the sample (Supplementary File S2, Figure S1).

At the provincial level, 97 out of 107 Italian provinces are represented by at least one response. Among the provinces with at least 20 responses, Turin is the most represented (140; 12.9%), followed by Brescia (62; 5.7%) and Bari (48; 4.4%). Foggia, Vicenza, Pordenone, and Cuneo each report 20 responses (1.8%).

#### 3.1.2. Professional Role

Most participants hold the role of clinical director and/or practice owner (550; 50.6%). Non-owner veterinarians employed within a practice account for 325 respondents (29.9%), while freelance veterinarians without a fixed practice location account for 213 respondents (19.6%) (Supplementary File S2, Figure S2).

#### 3.1.3. Training in Behavioral Medicine

Only 141 respondents (12.96%) report having obtained specific qualifications or training in behavioral medicine, whereas 947 (87.04%) have not undertaken structured training in this field (Supplementary File S2, Figure S3).

### 3.2. Clinical Experience and Management of Aggressive Cases (Questions 5–12)

Questions 5–12 were analyzed by thematic areas, including frequency of exposure to aggressive dogs, clinical management approaches, depth of history taking, and perception of the veterinarian’s preventive role.

#### 3.2.1. Frequency of Observation of Aggressive Behavior

Nearly all veterinarians reported having observed aggressive behavior during clinical procedures and having examined dogs with a history of previous aggression reported by the owner. Most respondents also reported frequent exposure to aggressive dogs during clinical activity, with more than 60% managing at least five aggressive cases per year and nearly one-third reporting more than 10 cases annually (Table 1; Supplementary File S2, Figures S4–S6).

#### 3.2.2. Owner Information and Specialist Referral

Most veterinarians reported routinely informing owners about the availability of training or behavioral support programs when faced with aggressive behavior. Referral to veterinarians with specific training in behavioral medicine represented the most frequently adopted approach in cases of suspected behavioral pathology, whereas referral to dog trainers or autonomous case management was less commonly reported (Table 1; Supplementary File S2, Figures S7 and S8).

#### 3.2.3. Depth of Domestic Behavioral History and Attention to Vulnerable Individuals

Most respondents reported investigating the behavioral history in the home environment when aggressive behavior was observed and assessing whether children had been educated on appropriate interactions with dogs in households with minors. In addition, a large majority recognized an active role for veterinarians in the prevention of canine aggression (Table 1; Supplementary File S2, Figures S9–S11).

### 3.3. Regulatory Awareness and Reporting Practices (Questions 13–17)

Questions 13–17 assessed awareness of the current regulatory framework and the use of reporting to ASL Veterinary Services. Slightly more than half of the respondents reported being aware of the Ministerial Ordinance of 6 August 2013, while awareness of the possibility of reporting to ASL Veterinary Services was somewhat higher. Despite this, only a minority of veterinarians reported having submitted at least one report, whereas most had never used this preventive tool (Table 2; Supplementary File S2, Figures S12–S14).

Inferential Analysis. The χ^2^ test revealed a statistically significant association between having specific training in behavioral medicine and awareness of the Ministerial Ordinance of 6 August 2013 (χ^2^ = 52.646; df = 1; *p* < 0.001). In particular, awareness of the Ordinance was more frequent among veterinarians with behavioral training compared to those without such training (83.0% vs. 50.3%). The effect size was moderate (Cramer’s V = 0.220).

A statistically significant association was also observed between awareness of the Ordinance and awareness of the possibility of reporting to ASL Veterinary Services (χ^2^ = 104.835; df = 1; *p* < 0.001). Awareness of the reporting tool was more frequent among veterinarians who reported being familiar with the Ordinance compared to those who were not (80.8% vs. 51.6%). In this case as well, the effect size was moderate (Cramer’s V = 0.310).

Finally, a statistically significant association was found between having specific training in behavioral medicine and having submitted at least one report to ASL Veterinary Services (χ^2^ = 33.989; df = 1; *p* < 0.001). Reporting was more frequent among veterinarians with behavioral training compared to those without such training (36.9% vs. 16.3%). The effect size was small but significant (Cramer’s V = 0.177). The results of the inferential analyses are summarized in Table 3 and presented in detail in Supplementary File S2, Figures S15–S17.

#### Multivariable Logistic Regression Analysis

**Figure 2:**
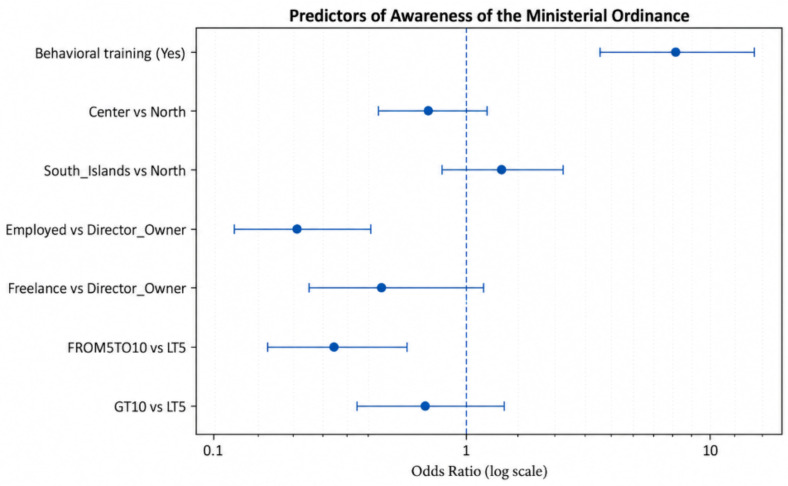
Forest plot of predictors of awareness of the Ministerial Ordinance among private veterinarians in Italy. Odds ratios (ORs) and 95% confidence intervals (CIs) are shown.

**Figure 3:**
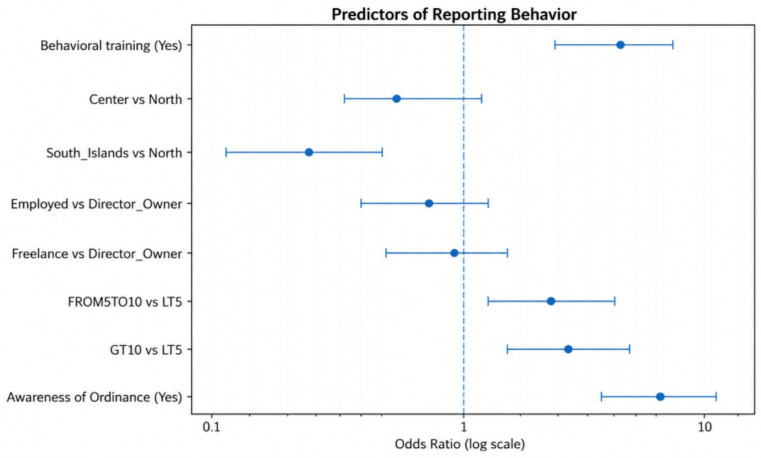
Forest plot of the multivariable logistic regression model assessing predictors of reporting behavior among private veterinarians in Italy. Odds ratios (ORs) and 95% confidence intervals (CIs) are shown.

To further investigate factors independently associated with regulatory awareness and reporting behavior, two multivariable binary logistic regression models were performed. In the first model, assessing predictors of awareness of the Ministerial Ordinance, specific training in behavioral medicine was independently associated with greater odds of regulatory awareness (OR = 4.82; 95% CI: 3.00–7.74; *p* < 0.001). Compared with practice owners/directors, employed veterinarians showed lower odds of awareness (OR = 0.59; 95% CI: 0.44–0.79; *p* < 0.001). Veterinarians reporting 5–10 aggressive dog encounters per year also showed lower odds of awareness compared with those reporting fewer than 5 encounters (OR = 0.68; 95% CI: 0.50–0.93; *p* = 0.016). Geographic macroarea was not significantly associated with awareness. In the second model, assessing predictors of reporting behavior, awareness of the Ministerial Ordinance was strongly associated with having submitted at least one report to ASL Veterinary Services (OR = 3.18; 95% CI: 2.18–4.62; *p* < 0.001). Specific training in behavioral medicine was also independently associated with reporting behavior (OR = 1.98; 95% CI: 1.29–3.02; *p* = 0.002). Veterinarians reporting more frequent encounters with aggressive dogs showed higher odds of reporting, particularly those reporting more than 10 encounters per year (OR = 1.95; 95% CI: 1.28–2.97; *p* = 0.002). Compared with veterinarians working in Northern Italy, respondents from Central and Southern Italy showed lower odds of reporting behavior (Table 4; Figure 2 and Figure 3).

Among the 206 respondents who had submitted a report, 114 (55.34%) were not aware of the outcome of the measure adopted. In 71 cases (34.47%), a behavioral rehabilitation program was imposed, while in 8 cases (3.88%), seizure of the animal was ordered. The “Other” category (13; 6.31%) includes: no measure taken (9), euthanasia (2), mandatory muzzle and leash (1), and home monitoring (1) (Figure S18). Among the 882 veterinarians who had not submitted reports or believed it was not within their responsibility, 393 (44.56%) had never considered it appropriate to proceed; 250 (28.37%) were not aware of the possibility of reporting; and 119 (13.48%) reported difficulties with the procedure. The “Other” category (120; 13.59%) includes: referral to a behavior specialist or provision of information on rehabilitation programs (47), fear of losing the client (17), lack of confidence in ASL operational procedures (15), fear of retaliation (9), report already submitted (5), concern about the dog’s fate (4), and belief of not being competent to manage the situation (3) (Supplementary File S2, Figure S19).

### 3.4. Risk Perception and Preventive Tools (Questions 18–23)

Questions 18–23 explored the perception of risk associated with specific dog types and the orientation toward structured preventive tools. Most veterinarians believed that all breeds included in the 2006 Ordinance may pose a potential risk if poorly managed, whereas a smaller proportion attributed risk only to selected breeds or excluded a specific breed-related risk (Table 5; Supplementary File S2, Figure S20).

Among the 310 detailed responses, the most frequently indicated breeds were the Pit Bull group, the Rottweiler, and the Dogo Argentino. These were followed by the Caucasian Shepherd Dog, the Central Asian Shepherd Dog, the Fila Brasileiro, and the Pit Bull Terrier (Supplementary File S2, Figure S21).

Among breeds not included in the Ordinance but considered potentially dangerous if poorly managed, the most frequently cited were the Czechoslovakian Wolfdog, the Maremma-Abruzzese Sheepdog, the Cane Corso, the German Shepherd, the Akita Inu, the American Staffordshire Terrier, and the Pit Bull Mastiff. These were followed by the Pyrenean Mastiff, the Rhodesian Ridgeback, and the Dobermann. Among the “Other” responses, some veterinarians indicated all breeds or mixed-breed dogs, whereas others referred more generally to medium–large or giant breeds; the Belgian Shepherd was also repeatedly cited (Supplementary File S2, Figure S22).

A large majority of respondents supported the reintroduction of mandatory owner training programs and favored the introduction of structured behavioral assessment protocols, most commonly beginning between 3 and 6 months of age with annual repetition (Table 5; Supplementary File S2, Figures S23 and S24).

Finally, almost all veterinarians supported the establishment of mandatory educational programs involving both dog and owner in the presence of identified risk factors (Table 5; Supplementary File S2, Figure S25).

## 4. Discussion

The findings of the present survey provide a nationwide overview of how small animal veterinarians in Italy perceive and implement their role in the prevention of dog aggression. In line with the aims of the study, the results show that veterinarians frequently encounter dogs displaying aggressive behavior and generally recognize an active preventive role for the profession. However, important gaps emerged between this perceived role and its practical implementation, particularly regarding awareness of the current regulatory framework and the use of reporting procedures to ASL Veterinary Services. The analyses further showed that specific training in behavioral medicine was independently associated with greater regulatory awareness and reporting behavior, while a strong consensus emerged in favor of structured preventive measures, including owner education and early behavioral assessment. Overall, these findings suggest that improving behavioral medicine training and strengthening the operational integration between private veterinarians and public veterinary services may represent important components of a more consistent national prevention strategy (Supplementary File S1, Question 1; Supplementary File S2, Figure S1).

Considering the recruitment strategy described in “Materials and Methods” section, based on institutional dissemination through provincial veterinary boards, this pattern should be interpreted as an indicator of participation in the survey rather than as a reflection of the territorial distribution of the profession. The observed regional differences are therefore consistent with varying levels of engagement in disseminating the communication and differing propensities to complete the questionnaire. The presence of respondents from all Italian regions, albeit with variable sample sizes, confirms the absence of territorial gaps in data collection. A similar interpretation applies to the provincial distribution (Supplementary File S1, Question 2).

With regard to the professional profile, the predominance of clinical directors and practice owners, alongside a substantial proportion of non-owner veterinarians and freelance practitioners without a fixed practice location, describes a sample that is heterogeneous with respect to the main modes of professional practice in the small animal practice (Supplementary File S1, Question 3; Supplementary File S2, Figure S2). The presence of professionals with a high degree of decision-making autonomy represents a relevant element, as these figures directly influence the organization of clinical procedures and the operational choices related to risk management.

Only 12.96% of veterinarians reported having received specific training in behavioral medicine (Supplementary File S1, Question 4; Supplementary File S2, Figure S3), indicating a limited diffusion of structured training pathways in this field. Open-ended responses confirmed this critical issue, extending it also to the period of undergraduate education as well.

This finding is consistent with the international literature. Kogan et al. reports that a substantial proportion of veterinarians consider the training received in behavioral medicine during their university education and clinical internships to be insufficient [25]. Similarly, Golden and Hanlon describe a widespread perception of inadequate academic preparation and identify the lack of specific competencies as a barrier in the clinical management of behavioral problems [26]. Lilly et al. further highlight particularly low baseline levels of knowledge, with significant improvements observed only following structured teaching, emphasizing the risk of insufficient “day one” competencies in the absence of comprehensive training [27].

More recent educational literature further supports the need for practical and clinically oriented training in veterinary behavioral medicine. Rametta et al. describe the introduction of clinical behavioral medicine activities using real clients and pets, reporting that students gained early experience in clinical communication, behavioral problems, and case write-ups. Their work also emphasizes that veterinary students need preparation to assess behavioral medicine issues, educate clients and staff, and incorporate early intervention for behavior problems into clinical practice [28].

This observation is particularly relevant in the Italian context, given the veterinarian’s role in the primary prevention of dog aggression. The early recognition of behavioral signals associated with potential risk to public safety requires specific competencies in clinical ethology, body language interpretation, and the assessment of aggressive motivations. This is consistent with the World Veterinary Association position statement, which emphasizes that veterinarians can contribute to the prevention and control of inappropriately aggressive dogs by educating owners, identifying inappropriate aggressive behavior early, communicating possible treatment recommendations, and referring to veterinary behavioral specialists when appropriate [29].

In the absence of widespread specialized training, it is plausible that the identification of at-risk situations and the activation of behavioral assessment pathways may be less consistent in clinical practice. This does not imply an individual deficiency, but rather highlights the need to strengthen the integration of behavioral medicine into undergraduate curricula and continuing education, as already suggested in the literature [25,26,27]. This interpretation is also coherent with broader developments in veterinary education, where competency-based frameworks emphasize the need to define, teach, assess, and document professional competencies across curricula, although implementation may be limited by resources, faculty development, assessment methods, and change-management barriers [30].

From an applied perspective, these findings support the relevance of structured educational programs aimed at improving veterinarians’ ability to recognize early behavioral warning signs, assess aggressive motivations, communicate risk to owners, and activate appropriate preventive pathways. This is also consistent with recent work on veterinary workplace clinical training, which emphasizes that preparedness is relevant because it directly affects performance and that the transition to competent veterinary practice depends on the integration and application of knowledge, skills, professional attributes, and clinical experience [31]. Such training may be particularly important for harmonizing clinical decision-making across different professional settings.

Nearly all veterinarians reported having observed aggressive behavior during clinical procedures (99.0%) and having frequently collected a history of previous aggression (96.1%), with more than 60% stating that they had examined at least five dogs showing aggressive behavior in the past year (Supplementary File S1, Questions 5–7; Supplementary File S2, Figures S4–S6).

The high prevalence of respondents reporting experience with aggressive behavior during clinical procedures is consistent with routine clinical practice, where veterinarians frequently encounter dogs showing fear-related or defensive aggression during handling. These findings support the interpretation of aggression as a recurrent component of the clinical caseload rather than an occasional event, reinforcing the role of the veterinarian as a frontline observer and potential sentinel for the early identification of at-risk situations.

As participation in the survey was voluntary, a degree of self-selection bias cannot be entirely excluded, with veterinarians more frequently exposed to aggressive cases or more interested in behavioral issues potentially being more inclined to participate. However, these factors should be considered when interpreting the findings, particularly the high levels of preventive awareness and behavioral sensitivity reported by respondents, and when assessing their generalizability to the broader population of Italian small animal veterinarians, although the large sample size and nationwide participation support the overall relevance of the observed trends.

This interpretation is further supported by the findings reported by Riemer et al. who describe the clinical setting as an environment in which aggressive behaviors may emerge clearly and repeatedly, thus providing a concrete opportunity for risk recognition and management [32]. The direct observation of aggression during the visit, especially when associated with a positive behavioral history, therefore assumes important signaling value from a preventive perspective.

Providing information to the owner represents a first level of operational response: 82% of veterinarians reported that they always provided information on training or support pathways, whereas 16% did so in cases considered severe (Supplementary File S1, Question 8; Supplementary File S2, Figure S7). These results indicate a widespread orientation toward owner responsibility.

As regards specialist referral, 81.34% referred the case to a veterinarian with specific training in behavioral medicine (Supplementary File S1, Question 9; Supplementary File S2, Figure S8), consistently with the limited diffusion of specialist training within the sample (Supplementary File S1, Question 4). This pattern appears to be partially at odds with the findings of Roshier and McBride, who reported that behavioral problems do not always lead to a structured pathway or to the involvement of specialist professionals [33].

Attention to the domestic context clearly emerges: 89% of veterinarians reported investigating the family history in the presence of aggression (Supplementary File S1, Question 10; Supplementary File S1, Figure S9). This pattern is consistent with national epidemiological data, according to which the most severe aggression episodes occur predominantly in domestic settings and involve owned dogs [2].

A similar proportion of veterinarians (82%) reported assessing whether children have been educated on appropriate interactions with dogs (Supplementary File S1, Question 11; Supplementary File S2, Figure S10). This attention is supported by the literature: in Italian cases of fatal dog attacks, preschool children represent one of the most affected groups [2]; international studies show that pediatric bites frequently occur in the home environment and involve familiar dogs [30], often with dynamics preceded by apparently harmless interactions [34]. The systematic integration of domestic history and the analysis of child–dog interactions therefore represents a central element in primary prevention [34,35].

Eighty percent of respondents recognized an active role for veterinarians in prevention (Supplementary File S1, Question 12; Supplementary File S2, Figure S11), in line with the literature identifying veterinarians as the first point of contact for behavioral problems and as key figures in the early identification of at-risk cases [26,36].

From a regulatory perspective, the Ministerial Ordinance of 6 August 2013 assigns a relevant role to veterinarians, both in informing owners and in the potential reporting to ASL Veterinary Services [17]. However, only 55% reported being aware of the Ordinance (Supplementary File S1, Question 13; Supplementary File S2, Figure S12), while 68% were aware of the possibility of reporting (Question 14, Figure S13). This discrepancy suggests that operational awareness is not always accompanied by a systematic reference to the regulatory framework.

Awareness of the regulatory framework was higher among veterinarians with behavioral medicine training (Supplementary File S2, Figures S15–S17), indicating an association between specialist training and greater alignment between regulation and practice. This finding, consistent with the inferential analyses, suggests that behavioral medicine training may promote not only the acquisition of clinical competencies but also greater awareness of the regulatory context and of the available operational tools, including the possibility of reporting to ASL Veterinary Services. This association remained significant after adjustment for geographic macroarea, professional role, and frequency of encounters with aggressive dogs, suggesting that behavioral medicine training independently contributes to regulatory awareness. The multivariable analysis further suggests that awareness of the regulatory framework itself may facilitate the activation of reporting procedures, independently of professional role or geographic area. This finding supports the interpretation that improving dissemination and understanding of the current Ordinance could contribute to a more consistent use of preventive tools in clinical practice.

The lower odds of regulatory awareness observed among veterinarians reporting 5–10 aggressive dog encounters per year compared with those reporting fewer than five encounters was unexpected and should be interpreted cautiously. This association did not show a clear dose–response pattern, as the effect was not observed among veterinarians reporting more than 10 encounters per year. Therefore, it may reflect sample distribution or residual confounding rather than a consistent inverse relationship between clinical exposure and regulatory awareness.

The barriers to reporting identified in the present study should also be interpreted within the current Italian regulatory framework. The Ministerial Ordinance of 6 August 2013 assigns veterinarians a role in informing owners and in signaling dogs requiring behavioral assessment in the interest of public safety; however, the regulation does not define standardized operational procedures nor impose mandatory reporting obligations in all situations considered at risk [17,18]. Consequently, the activation of preventive pathways may partially rely on individual professional judgment, potentially contributing to variability in reporting practices.

This aspect may be particularly relevant considering that previous evidence on fatal dog attacks in Italy showed that several dogs involved in severe or fatal aggression episodes had already displayed prior aggressive behavior toward humans or other animals [2]. Although the present study was not designed to evaluate the effectiveness of reporting procedures, these findings support the potential importance of early identification and preventive behavioral intervention in dogs already showing signs of aggression.

In this context, greater standardization of reporting procedures, improved communication between private veterinarians and ASL Veterinary Services, and continuing education initiatives focused on behavioral risk assessment may facilitate a more consistent implementation of preventive measures in clinical practice. These aspects may be particularly relevant in reducing uncertainty in clinical decision-making and in facilitating a more homogeneous implementation of preventive procedures across different territorial contexts. With regard to risk perception associated with breeds, 65.64% of respondents considered that all breeds included in the 2006 Ordinance may pose a risk if poorly managed (Question 18). This result is particularly relevant in the context of the debate on the possible reintroduction of breed-based regulatory approaches [37]. The international literature shows a similar position: while expressing opposition to breed-specific legislation, many veterinarians associate a higher risk with certain dog types, while recognizing the multifactorial nature of aggression [25,38].

It should be emphasized that the present study evaluated veterinarians’ perceptions of breed-related risk rather than objective epidemiological risk associated with specific breeds. Previous studies have shown that perceptions regarding specific dog breeds may be influenced not only by direct experience, but also by broader social and media narratives surrounding certain dog types and by the ongoing debate on breed-specific legislation [38,39]. Furthermore, breed attribution in aggression episodes presents important methodological limitations, particularly in the absence of genetic confirmation and in the frequent presence of mixed-breed dogs, which may contribute to inaccuracies in breed identification [38,40]. Accordingly, the present findings should not be interpreted as evidence supporting breed-specific risk per se, but rather as an expression of professional perceptions within the current Italian regulatory and social context.

The breeds most frequently indicated mainly belong to the functional groups of the Fédération Cynologique Internationale (FCI), selected for guarding and defense [41].

These results suggest that risk arises from the interaction between selective function, environmental management, and socialization, rather than to breed per se. Studies on livestock guardian dogs show that breeding practices and early socialization significantly influence the expression of aggression [42,43].

The orientation toward structured preventive tools is marked: the vast majority of respondents supported a mandatory training program for owners (Supplementary File S1, Question 21; Supplementary File S2, Figure S23) and the introduction of a mandatory behavioral assessment at a young age (Supplementary File S1, Question 22; Supplementary File S2, Figure S24). This approach is supported by evidence on the sensitive period of socialization, typically occurring between 3 and 12 weeks of age [44]. The proposal of an assessment between 3 and 6 months therefore appears to function primarily as a tool for early screening of individual risk. Although the present study was not designed to evaluate the effectiveness or feasibility of such interventions, the high level of agreement observed among respondents suggests that structured preventive pathways based on owner education and early behavioral assessment may be considered clinically relevant within the veterinary profession. These findings may support further investigation into the possible role of coordinated national prevention strategies focused on early risk identification and behavioral management.

Finally, the near-unanimous agreement on a mandatory educational pathway in the presence of risk factors (Supplementary File S1, Question 23; Supplementary File S2, Figure S25) consistently completed the preventive model outlined: primary prevention through training and screening, and secondary prevention through targeted interventions.

This study has some limitations. First, the predominant use of closed-ended questions (dichotomous or multiple-choice) may have reduced the granularity of the data collected, limiting the possibility of exploring certain aspects of the phenomenon in depth. In addition, the absence of validated scales for measuring behavioral and perceptual variables may affect the standardization and comparability of the results.

A further limitation is the self-reported nature of the data, which may introduce social desirability bias, with a possible overestimation of behaviors considered professionally appropriate. Moreover, participation was voluntary and dependent on dissemination by the provincial veterinary boards, potentially introducing self-selection bias and uneven geographical representation. Although geographic macroareas were included in the inferential analyses, the study was not designed to evaluate region-specific organizational differences in veterinary services or local implementation practices.

Finally, the cross-sectional design of the study does not allow causal relationships between the variables analyzed to be established. Accordingly, the multivariable associations observed in the logistic regression models should be interpreted as exploratory and associative rather than causal.

Despite these limitations, the large sample size and nationwide coverage contribute to providing a structured overview of professional practices and perceptions regarding the prevention of dog aggression in Italy.

## 5. Conclusions

The evidence obtained indicates that private veterinary practitioners may play a potentially central role in the prevention of dog aggression and in the protection of public safety, particularly through the early identification of at-risk situations in clinical and domestic contexts.

However, heterogeneity persists in both awareness and application of the current regulatory framework, highlighting a gap between the preventive role assigned to veterinarians and the operational tools actually used in clinical practice. In this context, training in behavioral medicine emerges as a key factor, independently associated with greater awareness of the regulatory framework and more frequent use of preventive tools.

The findings support the need to strengthen the integration of clinical, behavioral, and regulatory competencies in order to promote more consistent preventive approaches within the Italian professional context, particularly through clearer implementation pathways and improved collaboration between private practitioners and public veterinary services.

The results also suggest that aggression is generally perceived not as a breed-specific phenomenon, but rather as the outcome of multiple interacting factors related to the dog, its management, and the living environment, supporting the importance of individualized risk assessment.

Finally, the findings highlight the importance of strengthening behavioral medicine training during both undergraduate and postgraduate veterinary education in order to support more informed and effective management of behavioral risk.

## Figures and Tables

**Table 1:** Clinical encounters with aggressive dogs and preventive practices among private veterinarians in companion animal practice in Italy (*n* = 1088).

Variable	Category	n (%)
Observation of aggressive behavior during clinical procedures	Yes	1076 (99.0)
	No	12 (1.0)
History of aggression reported by owner	Yes	1046 (96.1)
	No	42 (3.9)
Aggressive cases managed in the past year	<5 cases	347 (31.9)
	5–10 cases	360 (33.1)
	>10 cases	322 (29.6)
	Do not recall	59 (5.4)
Provision of information on behavioral support	Always	896 (82.0)
	Only in severe cases	169 (16.0)
	Never or rarely	23 (2.0)
Referral in case of suspected behavioral pathology	Veterinarian with behavioral training	885 (81.3)
	Dog trainer	108 (9.9)
	Managed independently	29 (2.7)
	No further action	19 (1.8)
	Other	47 (4.3)
Assessment of behavioral history in domestic context	Yes	971 (89.0)
	No	117 (11.0)
Assessment of child–dog interaction (if children present)	Yes	890 (82.0)
	No	198 (18.0)
Perception of veterinarian’s preventive role	Yes	865 (80.0)
	No	55 (5.0)
	Uncertain	168 (15.0)

Data are presented as absolute numbers (n) and percentages (%). Percentages are calculated based on the total number of respondents. Behavioral support includes referral to veterinarians with specific training in behavioral medicine or other professional figures. Assessment of child–dog interaction in the presence of children.

**Table 2:** Discrepancy between perceived preventive role, regulatory awareness and reporting practices among private veterinarians (n = 1088).

Variable	Category	n (%)
Perception of veterinarian’s preventive role	Yes	865 (80.0)
	No	55 (5.0)
	Uncertain	168 (15.0)
Awareness of the Ministerial Ordinance (2013)	Yes	594 (55.0)
	No	494 (45.0)
Awareness of reporting to Veterinary Services	Yes	735 (68.0)
	No	353 (32.0)
Submission of reports to Veterinary Services	At least one report	206 (19.0)
	No reports	810 (74.0)
	Not my responsibility	72 (7.0)

Note: Data are presented as absolute numbers (n) and percentages (%).

**Table 3:** Associations between behavioral training, regulatory awareness and reporting practices among private veterinarians (n = 1088).

Variable	Category	n (%)
Behavioral training → Awareness of Ordinance	With training—Awareness	117 (83.0)
	With training—No awareness	24 (17.0)
	Without training—Awareness	477 (50.3)
	Without training—No awareness	470 (49.7)
Awareness of Ordinance → Awareness of reporting	Awareness—Awareness	480 (80.8)
	Awareness—No awareness	114 (19.2)
	No Awareness—Awareness	255 (51.6)
	No awareness—No awareness	239 (48.4)
Behavioral training → Reporting behavior	With training—At least one report	52 (36.9)
	With training—No report	89 (63.1)
	Without training—At least one report	154 (16.3)
	Without training—No report	793 (83.7)

Note: Data are presented as absolute numbers (n) and percentages (%). Percentages are calculated within each subgroup. All associations were statistically significant (χ^2^ test, *p* < 0.001; see Results for effect sizes). The symbol "→" indicates the association examined between the variables and does not imply causation.

**Table 4:** Multivariable logistic regression models assessing predictors of regulatory awareness and reporting behavior among private veterinarians in Italy.

Predictor	Model 1: Awareness of Ordinance OR (95% CI)	*p*-Value	Model 2: Reporting Behavior OR (95% CI)	*p*-Value
Behavioral training (Yes)	4.82 (3.00–7.74)	<0.001	1.98 (1.29–3.02)	0.002
Center vs. North	0.82 (0.61–1.10)	0.179	0.44 (0.30–0.66)	<0.001
South_Islands vs. North	1.28 (0.91–1.81)	0.157	0.24 (0.14–0.41)	<0.001
Employed vs. Director_Owner	0.59 (0.44–0.79)	<0.001	0.55 (0.36–0.82)	0.004
Freelance vs. Director_Owner	0.84 (0.59–1.18)	0.317	0.70 (0.45–1.09)	0.115
FROM5TO10 vs. LT5	0.68 (0.50–0.93)	0.016	1.57 (1.03–2.40)	0.034
GT10 vs. LT5	0.90 (0.65–1.24)	0.518	1.95 (1.28–2.97)	0.002
Awareness_Ordinance (Yes)	—	—	3.18 (2.18–4.62)	<0.001

Abbreviations: OR, odds ratio; CI, confidence interval; —, not applicable (predictor not included in the model). Model 1 assessed predictors of awareness of the Ministerial Ordinance of 6 August 2013. Model 2 assessed predictors of reporting behavior, defined as having submitted at least one report to ASL Veterinary Services. Reference categories were: no behavioral training, Northern Italy, director/owner role, fewer than 5 aggressive dog encounters per year, and no awareness of the Ministerial Ordinance (Model 2). Responses indicating “Do not recall” for aggressive dog encounters were treated as missing values and excluded from regression analyses.

**Table 5:** Attitudes toward preventive strategies and risk perception related to dog aggression among private veterinarians (n = 1088).

Variable	Category	n (%)
Support for mandatory owner training	Yes	978 (90.0)
	No	110 (10.0)
Preferred timing for behavioral assessment	3–6 months	639 (58.8)
	At adoption	192 (17.6)
	At 1 year	144 (13.3)
	Not useful	113 (10.3)
Support for mandatory education following risk assessment	Yes	1077 (99.0)
	No	11 (1.0)
Risk attribution to breeds (2006 Ordinance)	All breeds at risk if poorly managed	714 (65.6)
	Only some breeds	310 (28.5)
	No specific risk	64 (5.9)

Note: Data are presented as absolute numbers (n) and percentages (%). Percentages are calculated on the total number of respondents.

## Data Availability

The questionnaire used in this study is available at the following link: Google Forms questionnaire and in Supplementary File S1. The datasets generated and/or analyzed during the current study are available from the corresponding author upon reasonable request.

## Supplementary Materials

The following supporting information can be downloaded at: https://www.mdpi.com/article/10.3390/ani16111739/s1. Supplementary File S1: Full questionnaire administered to Italian small animal veterinarians (Italian and English versions). Supplementary File S2: Supplementary Figures S1–S25 illustrating the distribution of survey responses, associations between behavioral training, awareness of the Ordinance and reporting practices, risk perception of dog breeds, and preventive strategies for dog aggression management.
