# Supplementary material for: Small Animal Veterinarians’ Perceptions and Practices in Dog Aggression Prevention in Italy: A National Survey
Source: Animals (Basel). 2026 Jun 5;16(11):1739. doi: 10.3390/ani16111739 (PMC13256073; doi:10.3390/ani16111739)
Supplement: Supplementary file 1 [file animals-16-01739-s001.zip › Supplementary File S1.pdf]

STRUTTURA DEL QUESTIONARIO

→SEZIONE PRELIMINARE: CONSENSO INFORMATO ALLA PARTECIPAZIONE ALLA RICERCA

Ai sensi dell'art. 13 del Regolamento (UE) 2016/679, La informiamo che:
i dati raccolti saranno elaborati in modo manuale e/o elettronico ed utilizzati, unicamente per gli obiettivi della ricerca stessa, solo in forma aggregata attraverso la predisposizione di tabelle, indici e grafici, senza fare riferimento, in alcun modo, ai singoli soggetti intervistati. I risultati della ricerca saranno pubblicati in forma riassuntiva e in nessun caso eventuali brevi citazioni saranno riconducibili a singole persone. I dati raccolti sono totalmente anonimi e non verranno usati a scopo di lucro. Pertanto, si senta libero di rispondere al questionario o di rifiutare la compilazione in qualsiasi momento lei voglia.

DICHIARO

- di aver compreso che la partecipazione allo studio è del tutto volontaria e libera, che ci si potrà ritirare dallo studio in qualsiasi momento, senza dover dare spiegazioni e senza che ciò comporti alcuno svantaggio o pregiudizio;
- di aver compreso la natura e le attività che la partecipazione allo studio comporta e i relativi eventuali rischi;
- di aver compreso che la partecipazione a questo studio non comporterà il riconoscimento di alcun vantaggio di natura economica diretto o indiretto.

Q0: *Conseguentemente, può scegliere se partecipare allo studio, nella consapevolezza che tale consenso è manifestato liberamente ed è revocabile in ogni momento senza che ciò comporti alcuno svantaggio o pregiudizio.*

a) Ho compreso ed acconsento
b) Ho compreso e NON acconsento

→SEZIONE 1: PROFILO PROFESSIONALE

Q1: *Indichi la regione in cui svolge l'attività veterinaria*
(Elenco di tutte le province italiane)

Q2: *Indichi la provincia in cui svolge l'attività veterinaria*
(Elenco di tutte le regioni italiane)

Q3: *Qual è la sua area di pertinenza?*

NOTA IMPORTANTE: *Questo studio è rivolto esclusivamente a medici veterinari che si occupano di piccoli animali.*

Qualora dichiari di ***non occuparsi di piccoli animali****, il questionario terminerà automaticamente, poiché non è necessario proseguire con la compilazione.*

a) *Veterinario libero professionista*
b) *Veterinario ASL*
c) *Altro*

Q4: *Ha conseguito titoli di studio o formazione specifica in Medicina Comportamentale?*

a) Si
b) No

→SEZIONE 2: ESPERIENZA CLINICA CON AGGRESSIVITÀ

Q5: *Le è mai capitato di visitare cani che manifestano comportamenti aggressivi durante le procedure cliniche?*

a) Si
b) No

Q6: *Le è mai capitato di visitare cani con storia riferita dal proprietario di comportamenti aggressivi pregressi?*

a) Si
b) No

Q7: *Quanti cani con comportamenti aggressivi le è capitato di visitare nell'ultimo anno?*

a) Meno di 5
b) 5-10
c) Più di 10
d) Non ricordo

Q8: *Quando visita un cane che manifesta comportamenti aggressivi, informa abitualmente il proprietario sull'esistenza di percorsi formativi o di supporto alla gestione comportamentale?*

a) Si, sempre
b) Si, ma solo nei casi che ritengo gravi
c) No

Q9: *In caso di sospetto di patologia comportamentale, a quale figura professionale riferisce il caso?*

a) Medico veterinario perfezionato in Medicina Comportamentale
b) Istruttore Cinofilo
c) Non lo riferisco, lo gestisco in autonomia
d) Mi limito a gestire il problema di presentazione (cioè il motivo per cui l'animale è stato portato a visita), senza approfondire l'aspetto comportamentale.
e) Altro

Policlinico di Bari
P.zza Giulio Cesare, 11
70124 Bari (Italy)

Coordinamento del Dipartimento
Tel. +39 0805478627
e-mail: delfinamaria.misuraca@uniba.it.

Q10: *In caso di comportamenti aggressivi riscontrati durante la visita clinica, approfondisce l'anamnesi comportamentale del cane in ambiente domestico (rapporti con i membri della famiglia e/o con altri animali)?*

a) Si
b) No

Q11: *In presenza di bambini nel nucleo familiare, chiede se questi sono stati adeguatamente educati a un corretto approccio al cane?*

a) Si
b) No

→SEZIONE 3: ASPETTI NORMATIVI E DECISIONALI

Q12: *Ritiene che il veterinario debba avere un ruolo attivo nella prevenzione delle aggressioni?*

a) Si
b) No
c) Non saprei

Q13: *È a conoscenza dell'Ordinanza Ministeriale del 6 agosto 2013 (tuttora in vigore), che disciplina la tutela dell'incolumità pubblica dalle aggressioni canine, e che richiama specifiche norme del Codice Penale (artt. 544-ter e 650) e la Legge quadro 281/1991 sugli animali da affezione?*

a) Si
b) No

Q14: *È a conoscenza del fatto che, ai sensi della normativa vigente, in presenza di un cane con marcati segni di aggressività, il medico veterinario può effettuare segnalazione all'ASL competente?*

a) Si
b) No

Q15: *In seguito alla valutazione clinica di un cane ritenuto gravemente aggressivo e potenzialmente pericoloso per l'incolumità pubblica, ha mai effettuato una segnalazione ai servizi veterinari dell'ASL competente?*

a) Si
b) No
c) Non era di mia competenza

Q16: *In caso di segnalazione effettuata, quale provvedimento è stato adottato dall'ASL competente?*

a) Sequestro dell'animale
b) Obbligo per il proprietario di seguire un percorso di rieducazione comportamentale per il cane
c) Provvedimento non noto
d) Altro

Q17: *Se non ha mai fatto la segnalazione, per quale motivo?*

a) Non ero a conoscenza della possibilità di farlo
b) Non è facile capire la procedura da seguire
c) Non l'ho mai ritenuto opportuno
d) Altro

→SEZIONE 4: PERCEZIONE DEL RISCHIO E STRATEGIE DI PREVENZIONE

Q18: *Secondo lei, le razze e/o incroci di razze indicate nella precedente Ordinanza del 2006 costituiscono un pericolo per l'incolumità pubblica se mal gestite?*

a) Si, tutte quelle incluse nell'elenco
b) No, solo alcune
c) Nessuna delle precedenti

Di seguito, trova elencate in ordine alfabetico le razze e/o incroci di razze comprese nell'Ordinanza sopra menzionata:

- American Bulldog;
- Cane da pastore di Charplanina;
- Cane da pastore dell'Anatolia;
- Cane da pastore dell'Asia centrale;
- Cane da pastore del Caucaso;
- Cane da Serra da Estreilla;
- Dogo Argentino;
- Fila brazileiro;
- Perro da canapo majoero;
- Perro da presa canario;
- Perro da presa Mallorquin;
- Pit bull; Pit bull mastiff;

- Pit bull terrier;
- Rafeiro do alentejo;
- Rottweiler;
- Tosa inu.

Q19: Indichi nello specifico quale/i razze e/o incroci di razze dell'elenco proposto nell'Ordinanza 2006 ritiene potenzialmente pericolose.
Le voci sono presentate in ordine alfabetico. (Selezione multipla)

a) American Bulldog
b) Cane da pastore di Charplanina
c) Cane da pastore dell'Anatolia
d) Cane da pastore dell'Asia centrale
e) Cane da pastore del Caucaso
f) Cane da Serra da Estreilla
g) Dogo Argentino
h) Fila brazileiro
i) Perro da canapo majoero
j) Perro da presa canario
k) Perro da presa Mallorquin
l) Pit bull; Pit bull mastiff
m) Pit bull terrier
n) Rafeiro do alentejo
o) Rottweiler
p) Tosa inu

Q20: Di seguito sono elencate altre razze e/o incroci di razze di taglia medio-grande, non incluse nell'Ordinanza, che potrebbero comunque costituire un rischio se mal gestite. Ritiene che alcune di queste siano potenzialmente pericolose?
Selezioni una o più opzioni tra quelle proposte. Se non ritiene nessuna delle razze indicate potenzialmente pericolosa, può lasciare la domanda vuota e proseguire con la compilazione del questionario.

a) Akita Inu
b) Alano
c) Alaskan Malamute
d) American Bully
e) American Staffordshire Terrier
f) Australian Shepherd (Pastore Australiano)
g) Beauceron
h) Border Collie
i) Bovaro del Bernese

j) Boxer
k) Bracco Italiano
l) Bullmastiff
m) Cane Corso
n) Cane Lupo Cecoslovacco
o) Continental Bulldog
p) Dalmata
q) Dobermann
r) Dogue de Bordeaux
s) Golden Retriever
t) Irish Wolfhound
u) Komondor
v) Kuvasz
w) Labrador Retriever
x) Mastino dei Pirenei
y) Mastino Spagnolo
z) Pastore Maremmano-Abruzzese
aa) Pastore Tedesco
bb) Pit Bull Mastiff
cc) Pointer
dd) Rhodesian Ridgeback
ee) San Bernardo
ff) Setter Inglese
gg) Spinone Italiano
hh) Staffordshire Bull Terrier
ii) Terranova
jj) Weimaraner
kk) Working Pit Bulldog
ll) Altro

Q21: *Per le razze e/o incroci di razze da Lei indicate nelle domande precedenti, sarebbe favorevole all'introduzione di un percorso formativo obbligatorio per il proprietario, simile a quanto previsto in passato con il cosiddetto "patentino" di cui si era prospettata l'introduzione con l'Ordinanza Ministeriale del 12 dicembre 2006?*

a) Si
b) No

Q22: *Per le razze e/o incroci da Lei selezionati, sarebbe favorevole all'introduzione di una visita comportamentale obbligatoria, al fine di certificare che il cane sia ben gestito e non rappresenti un rischio per la sicurezza pubblica?*

a) Sì, al momento dell'adozione
b) Sì, a partire dai 36 mesi e da ripetere con cadenza annuale
c) Si, al compimento del primo anno
d) No, non la ritengo utile

Q23: *Nel caso in cui, a seguito di una visita comportamentale obbligatoria, emergessero elementi che indicano un rischio potenziale per la sicurezza pubblica, sarebbe favorevole a rendere obbligatorio un percorso educativo rivolto al cane e al suo proprietario?*

a) Si
b) No

→SEZIONE 5: COMMENTI LIBERI

Q24: *Se desidera, può aggiungere considerazioni personali su quanto emerso o proposte operative: (Risposta aperta facoltativa)*

QUESTIONNAIRE STRUCTURE

Policlinico di Bari
P.zza Giulio Cesare, 11
70124 Bari (Italy)

Coordinamento del Dipartimento
Tel. +39 0805478627
e-mail: delfinamaria.misuraca@uniba.it.

PRELIMINARY SECTION: INFORMED CONSENT TO PARTICIPATE IN THE RESEARCH

Pursuant to Article 13 of Regulation (EU) 2016/679, you are hereby informed that:
the data collected will be processed manually and/or electronically and used solely for the purposes of the research itself, only in aggregated form through the preparation of tables, indices, and graphs, without making any reference to individual respondents. The research results will be published in summary form, and in no case will any brief citations be traceable to individual persons. The data collected are entirely anonymous and will not be used for profit. Therefore, please feel free to respond to the questionnaire or to decline its completion at any time you wish.

I DECLARE

- that I have understood that participation in the study is entirely voluntary and free; that I may withdraw from the study at any time, without having to provide any explanation and without any disadvantage or prejudice;
- that I have understood the nature of the activities that participation in the study entails and any related risks;
- that I have understood that participation in this study will not entail any direct or indirect economic benefit.

Q0: *Consequently, you may choose whether to participate in the study, in the awareness that such consent is freely expressed and may be revoked at any time without entailing any disadvantage or prejudice.*

a) I have understood and consent
b) I have understood and do NOT consent

→SECTION 1: PROFESSIONAL PROFILE

Q1: *Please indicate the region in which you practice as a veterinarian*
(List of all Italian regions)

Q2: *Please indicate the province in which you practice as a veterinarian*
(List of all Italian provinces)

Q3: *What is your area of practice?*

***IMPORTANT NOTE**: This study is addressed exclusively to veterinarians who deal with small animals.*
*If you indicate that you **do not deal with small animals**, the questionnaire will end automatically, as it is not necessary to continue with its completion.*

a) Private Veterinarian working in the companion animal sector
b) ASL (Local Health Authority) veterinarian
c) Other

Q4: Have you obtained specific qualifications or training in Behavioral Medicine?
a) Yes
b) No

→SECTION 2: CLINICAL EXPERIENCE WITH AGGRESSION

Q5: Have you ever examined dogs that displayed aggressive behavior during clinical procedures?
a) Yes
b) No

Q6: Have you ever examined dogs with an owner-reported history of previous aggressive behavior?
a) Yes
b) No

Q7: How many dogs with aggressive behavior have you examined over the past year?
a) Fewer than 5
b) 5–10
c) More than 10
d) I do not remember

Q8: When examining a dog that displays aggressive behavior, do you routinely inform the owner about the existence of training programs or behavioral management support?
a) Yes, always
b) Yes, but only in cases I consider severe
c) No

Q9: *In case of suspected behavioral pathology, to which professional figure do you refer the case?*

a) Veterinarian with advanced training in Behavioral Medicine
b) Dog trainer
c) I do not refer the case; I manage it independently
d) I limit myself to managing the presenting problem (i.e., the reason the animal was brought in for examination), without further investigating the behavioral aspect.
e) Other

Q10: *In the case of aggressive behavior observed during the clinical visit, do you investigate the dog's behavioral history in the home environment (relationships with family members and/or other animals)?*

a) Yes
b) No

Q11: *When children are present in the household, do you ask whether they have been adequately educated about a correct approach to the dog?*

a) Yes
b) No

→SECTION 3: REGULATORY AND DECISION-MAKING ASPECTS

Q12: *Do you believe that the veterinarian should play an active role in the prevention of dog aggression?*

a) Yes
b) No
c) I am not sure

Q13: *Are you aware of the Ministerial Ordinance of 6 August 2013 (still in force), which regulates the protection of public safety from canine aggression, and which refers to specific provisions of the Italian Criminal Code (Articles 544-ter and 650) and to Framework Law 281/1991 on companion animals?*

a) Yes
b) No

Q14: *Are you aware that, under current regulations, when a dog shows marked signs of aggression, the veterinarian may submit a report to the competent ASL (Local Health Authority)?*

a) Yes
b) No

Q15: *Following the clinical assessment of a dog deemed severely aggressive and potentially dangerous to public safety, have you ever filed a report with the veterinary services of the competent ASL?*

a) Yes
b) No
c) It was not within my responsibility

Q16: If a report was submitted, what measure was adopted by the competent ASL?

a) Seizure of the animal
b) Obligation for the owner to undertake a behavioral rehabilitation program for the dog
c) Measure unknown
d) Other

Q17: If you have never submitted a report, what was the reason?

a) I was not aware that I could do so
b) The procedure to follow is not easy to understand
c) I have never considered it appropriate
d) Other

→SECTION 4: RISK PERCEPTION AND PREVENTION STRATEGIES

Q18: In your opinion, do the breeds and/or breed mixes listed in the previous 2006 Ordinance pose a danger to public safety if poorly managed?

a) Yes, all of those included in the list
b) No, only some of them
c) None of the above

Below, listed in alphabetical order, are the breeds and/or breed mixes included in the aforementioned Ordinance:

- American Bulldog;
- Šarplaninac (Charplanina Shepherd Dog);
- Anatolian Shepherd Dog;
- Central Asian Shepherd Dog;
- Caucasian Shepherd Dog;
- Estrela Mountain Dog (Cão da Serra da Estrela);
- Dogo Argentino;
- Fila Brasileiro;
- Perro de Pastor Majorero;
- Perro de Presa Canario;
- Perro de Presa Mallorquín;
- Pit Bull; Pit Bull Mastiff;

- Pit Bull Terrier;
- Rafeiro do Alentejo;
- Rottweiler;
- Tosa Inu.

Policlinico di Bari
P.zza Giulio Cesare, 11
70124 Bari (Italy)

Coordinamento del Dipartimento
Tel. +39 0805478627
e-mail: delfinamaria.misuraca@uniba.it.

Q19: Please specifically indicate which breed(s) and/or breed mix(es) from the list proposed in the 2006 Ordinance you consider potentially dangerous.
Items are listed in alphabetical order. (Multiple-choice question)

a) American Bulldog
b) Šarplaninac (Charplanina Shepherd Dog)
c) Anatolian Shepherd Dog
d) Central Asian Shepherd Dog
e) Caucasian Shepherd Dog
f) Estrela Mountain Dog (Cão da Serra da Estrela)
g) Dogo Argentino
h) Fila Brasileiro
i) Perro de Pastor Majorero
j) Perro de Presa Canario
k) Perro de Presa Mallorquín
l) Pit Bull; Pit Bull Mastiff
m) Pit Bull Terrier
n) Rafeiro do Alentejo
o) Rottweiler
p) Tosa Inu

Q20: Listed below are other medium- to large-sized breeds and/or breed mixes, not included in the Ordinance, that could nevertheless pose a risk if poorly managed. Do you consider any of these potentially dangerous?
Please select one or more of the options proposed. If you consider none of the listed breeds potentially dangerous, you may leave the question blank and continue with the questionnaire.

a) Akita Inu
b) Great Dane
c) Alaskan Malamute
d) American Bully
e) American Staffordshire Terrier
f) Australian Shepherd
g) Beauceron
h) Border Collie
i) Bernese Mountain Dog
j) Boxer

Policlinico di Bari
P.zza Giulio Cesare, 11
70124 Bari (Italy)

Coordinamento del Dipartimento
Tel. +39 0805478627
e-mail: delfinamaria.misuraca@uniba.it.

k) Bracco Italiano (Italian Pointer)
l) Bullmastiff
m) Cane Corso
n) Czechoslovakian Wolfdog
o) Continental Bulldog
p) Dalmatian
q) Dobermann
r) Dogue de Bordeaux
s) Golden Retriever
t) Irish Wolfhound
u) Komondor
v) Kuvasz
w) Labrador Retriever
x) Pyrenean Mastiff
y) Spanish Mastiff
z) Maremma-Abruzzese Sheepdog
aa) German Shepherd
bb) Pit Bull Mastiff
cc) Pointer
dd) Rhodesian Ridgeback
ee) Saint Bernard
ff) English Setter
gg) Spinone Italiano
hh) Staffordshire Bull Terrier
ii) Newfoundland
jj) Weimaraner
kk) Working Pit Bulldog
ll) Other

Q21: *For the breeds and/or breed mixes you indicated in the previous questions, would you be in favor of the introduction of a mandatory training course for the owner, similar to what was previously envisaged with the so-called "patentino" (owner license) whose introduction had been proposed under the Ministerial Ordinance of 12 December 2006?*

a) Yes

b) No

Q22: *For the breeds and/or breed mixes you selected, would you be in favor of the introduction of a mandatory behavioral examination, in order to certify that the dog is properly managed and does not represent a risk to public safety?*

a) Yes, at the time of adoption
b) Yes, starting at 36 months of age and to be repeated annually
c) Yes, upon the completion of the first year of age
d) No, I do not consider it useful

Q23: *If, following a mandatory behavioral examination, elements emerged indicating a potential risk to public safety, would you be in favor of making mandatory an educational program addressed to both the dog and its owner?*

a) Yes
b) No

→SECTION 5: FREE COMMENTS

Q24: If you wish, you may add personal considerations on the issues raised or any operational suggestions:
(Optional open-ended response)

Policlinico di Bari
P.zza Giulio Cesare, 11
70124 Bari (Italy)

Coordinamento del Dipartimento
Tel. +39 0805478627
e-mail: delfinamaria.misuraca@uniba.it.
