# Supplementary material for: Small Animal Veterinarians’ Perceptions and Practices in Dog Aggression Prevention in Italy: A National Survey
Source: Animals (Basel). 2026 Jun 5;16(11):1739. doi: 10.3390/ani16111739 (PMC13256073; doi:10.3390/ani16111739)
Supplement: Supplementary file 1 [file animals-16-01739-s001.zip › Supplementary File S2.pdf]

SUPPLEMENTARY FIGURES: SURVEY RESULTS ON VETERINARY PRACTICES, REGULATORY KNOWLEDGE, AND PREVENTION OF DOG AGGRESSION IN ITALY

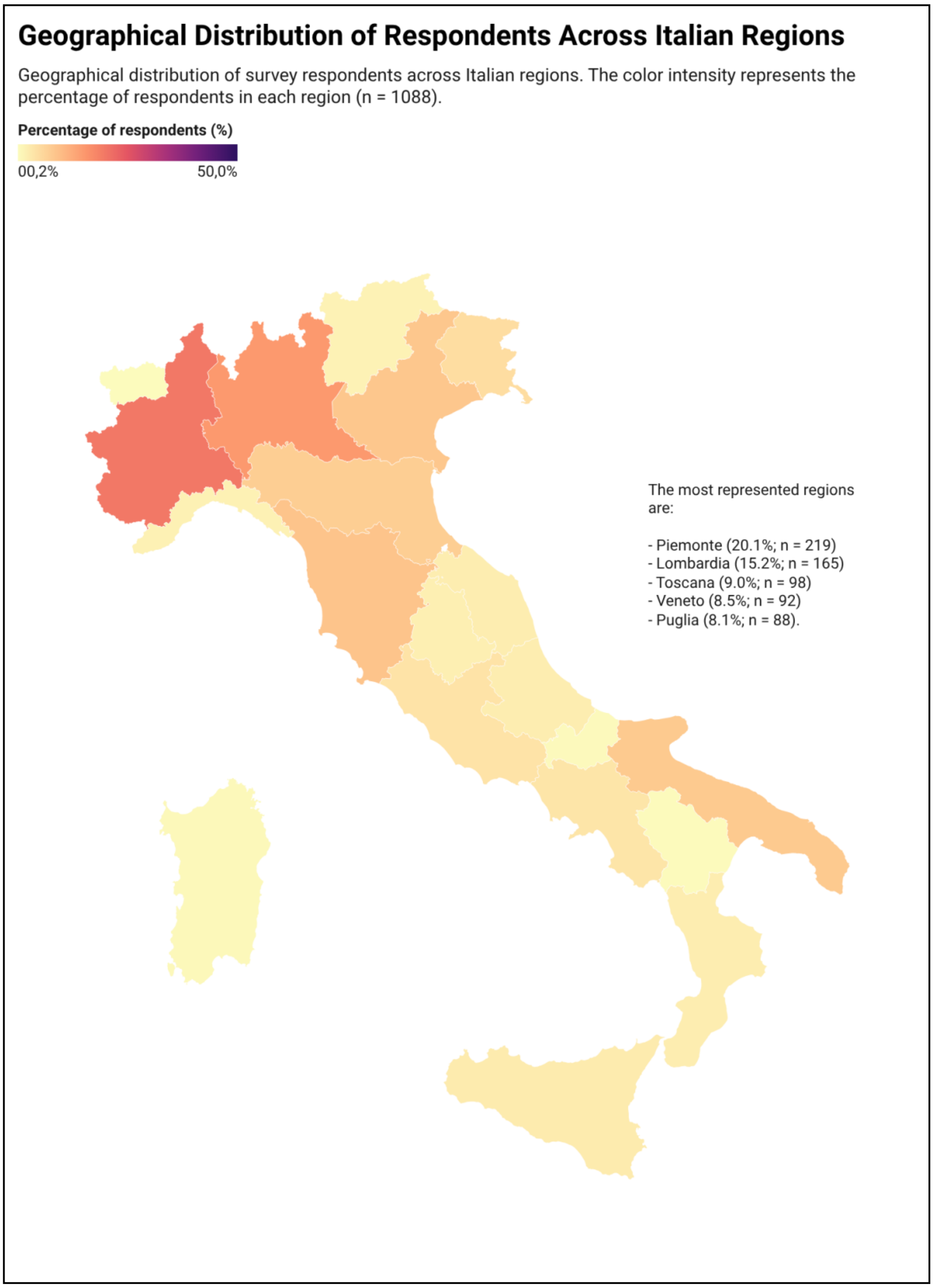

Figure S1. Geographical distribution of survey respondents across Italian regions. Color intensity represents the percentage of respondents in each region (n = 1,088). The most represented regions are Piemonte (20.1%; n = 219), Lombardia (15.2%; n = 165), Toscana (9.0%; n = 98), Veneto (8.5%; n = 92), and Puglia (8.1%; n = 88).

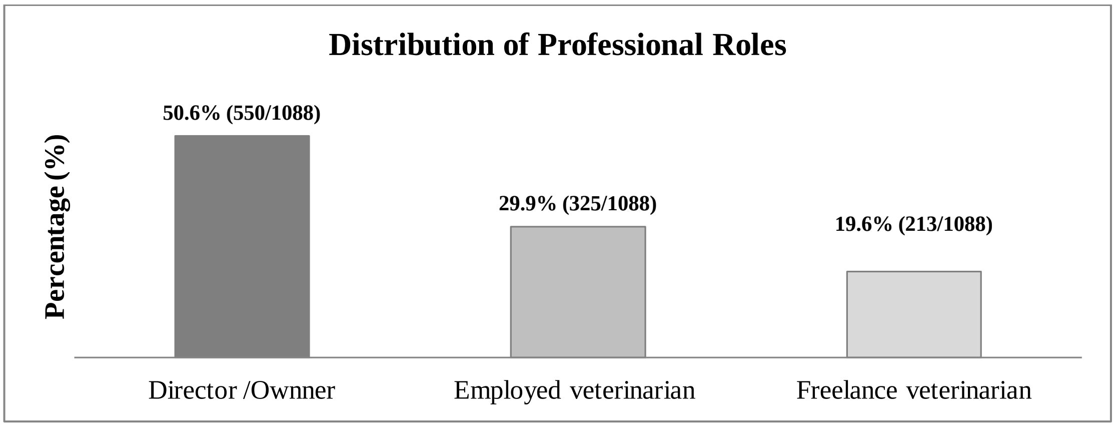

Figure S2. Distribution of respondents by professional role. Most respondents were clinical directors and/or practice owners (50.6%; 550/1088), followed by employed veterinarians (29.9%; 325/1088) and freelance veterinarians (19.6%; 213/1088).

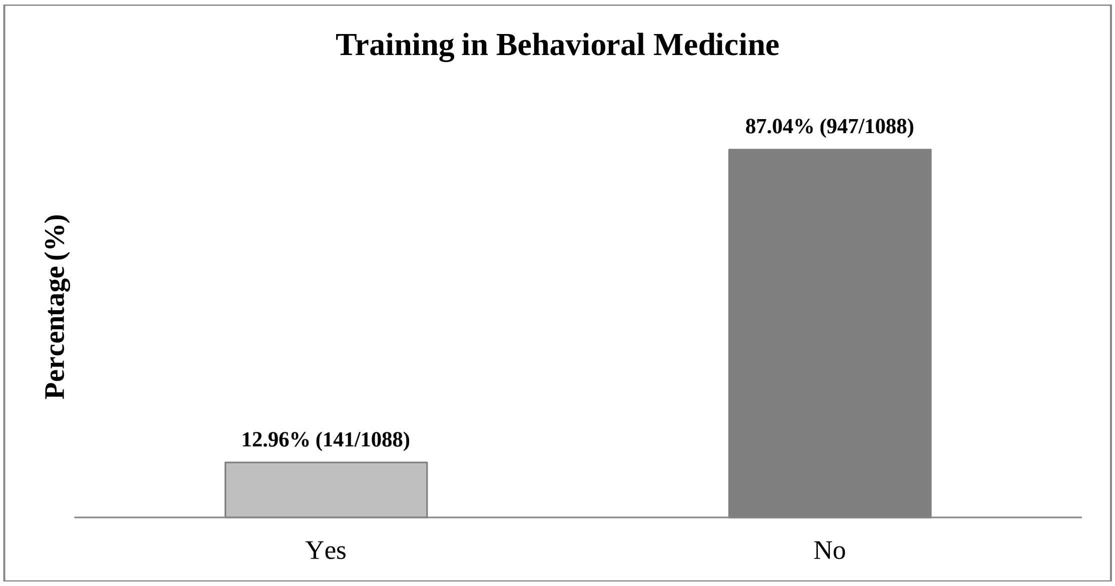

Figure S3. Distribution of respondents by training in behavioral medicine. A total of 12.96% of respondents (141/1088) reported having received training in behavioral medicine, whereas 87.04% (947/1088) had not.

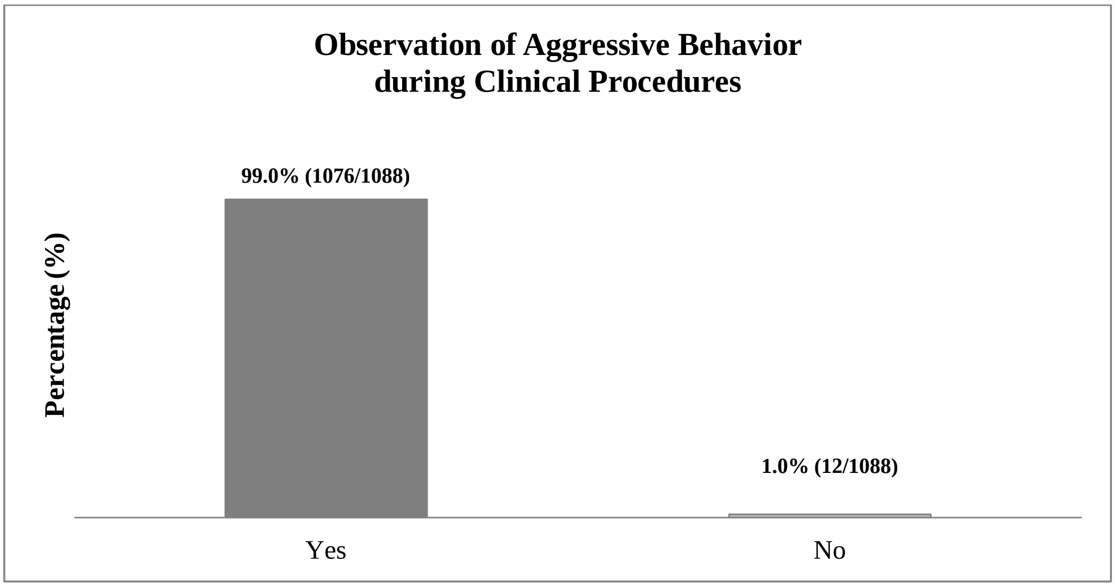

Figure S4. Observation of aggressive behavior during clinical procedures. A total of 99.0% of respondents (1076/1088) reported having observed aggressive behavior during clinical procedures, whereas 1.0% (12/1088) reported no such experience.

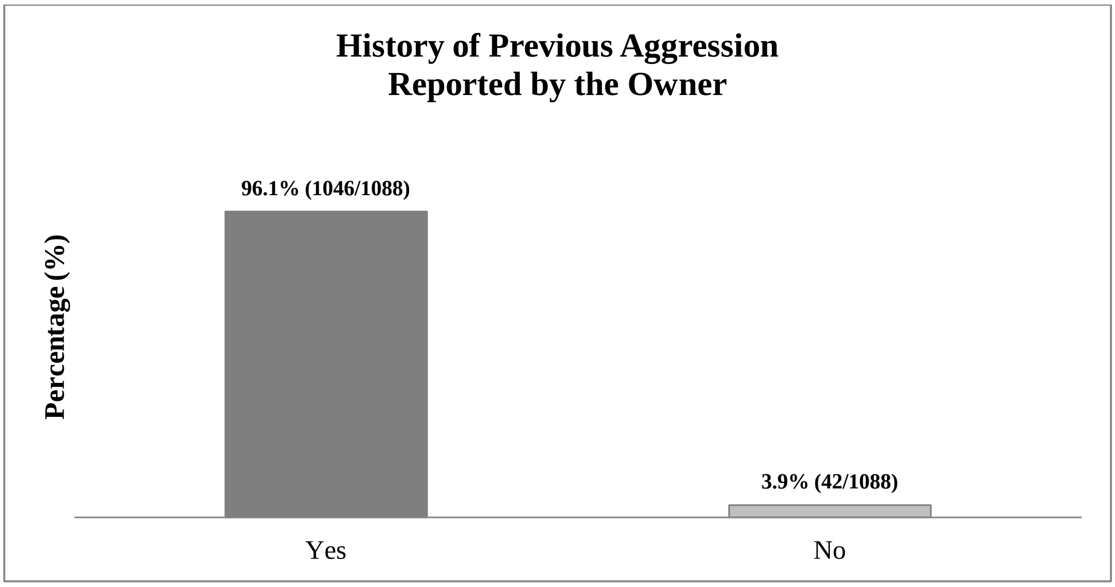

Figure S5. History of previous aggression reported by the owner. A total of 96.1% of respondents (1046/1088) reported having examined dogs with a history of previous aggression as reported by the owner, whereas 3.9% (42/1088) had not.

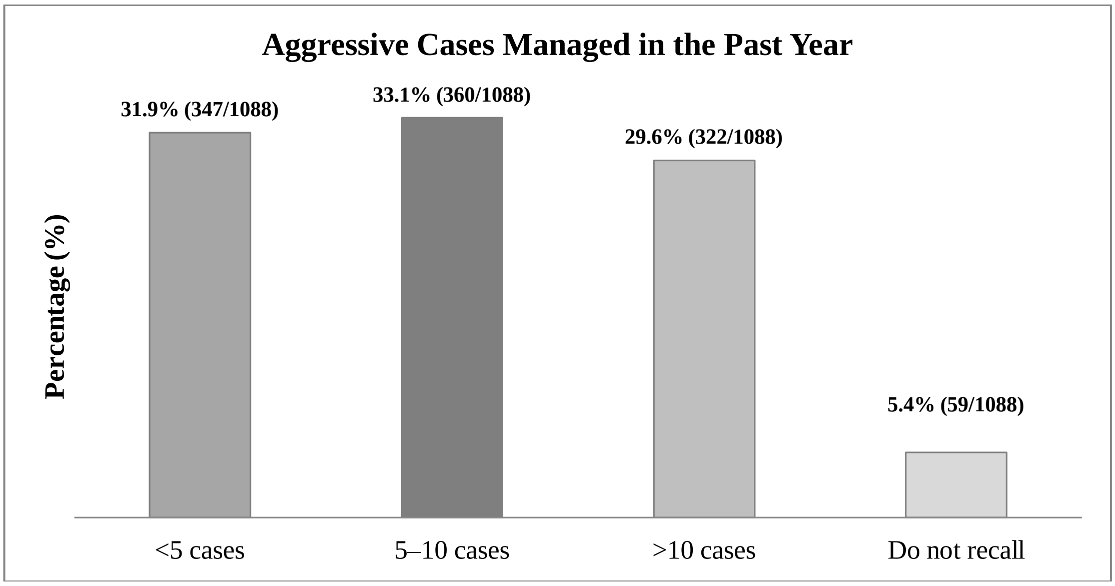

Figure S6. Aggressive cases managed in the past year. A total of 31.9% of respondents (347/1088) reported managing fewer than 5 cases, 33.1% (360/1088) between 5 and 10 cases, and 29.6% (322/1088) more than 10 cases, whereas 5.4% (59/1088) did not recall the number of cases managed.

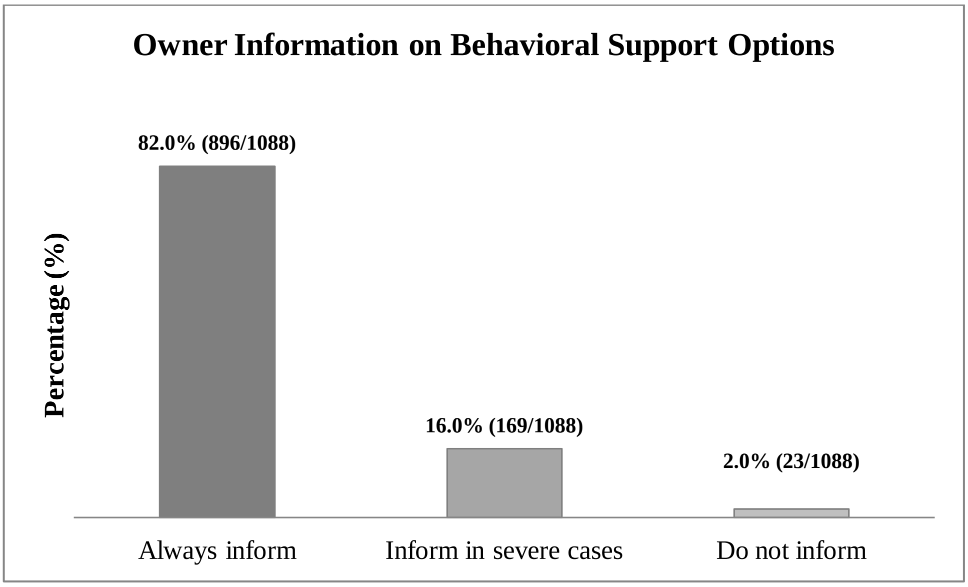

Figure S7. Owner information on behavioral support options. A total of 82.0% of respondents (896/1088) reported always informing owners about behavioral support options, 16.0% (169/1088) reported doing so only in severe cases, whereas 2.0% (23/1088) reported not providing such information.

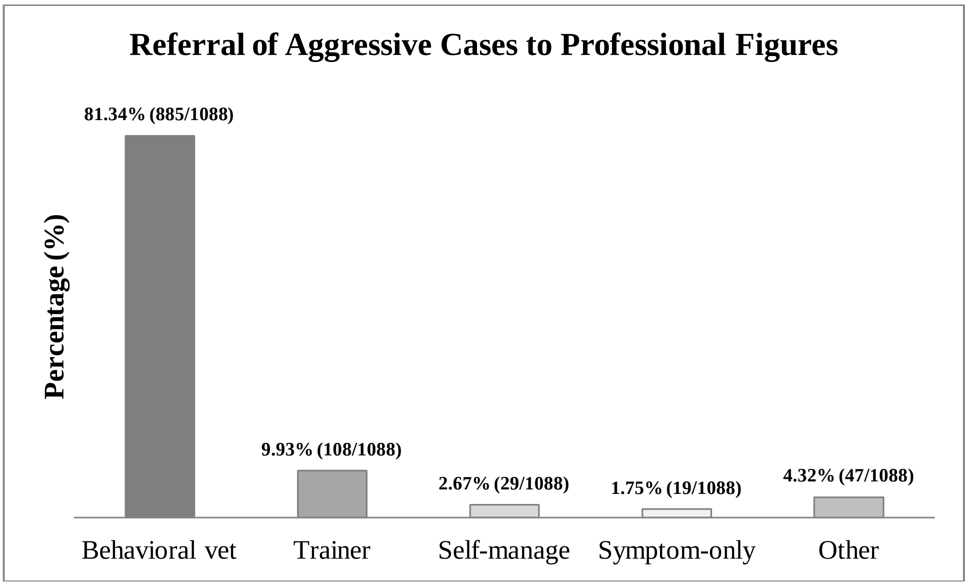

Figure S8. Referral of aggressive cases to professional figures. A total of 81.34% of respondents (885/1088) reported referring cases to a veterinarian with specific training in behavioral medicine, 9.93% (108/1088) to a dog trainer, 2.67% (29/1088) reported managing the case independently, and 1.75% (19/1088) reported limiting their intervention to the presenting problem, whereas 4.32% (47/1088) selected other options.

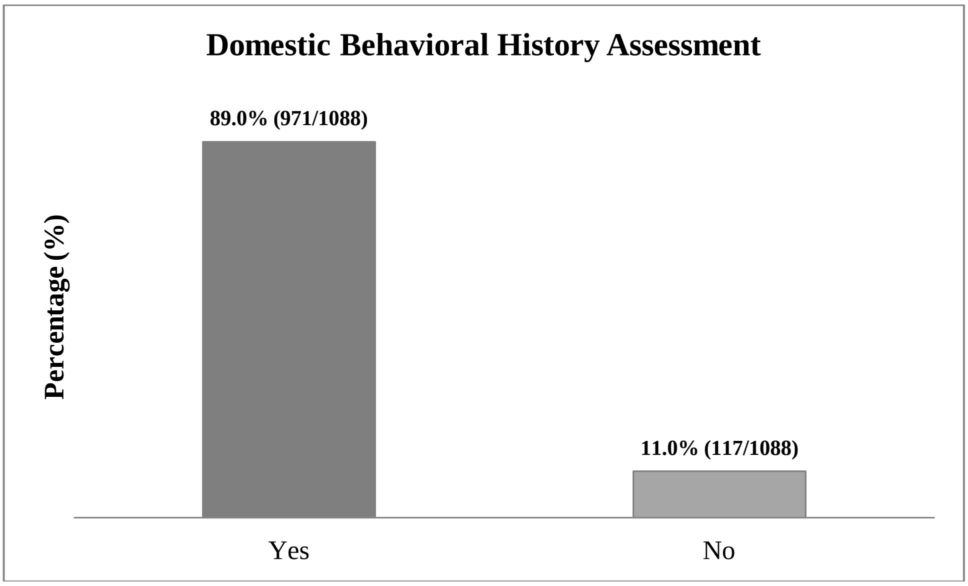

Figure S9. Assessment of behavioral history in the domestic context when aggressive behavior is observed. A total of 89.0% of respondents (971/1088) reported investigating the behavioral history in the domestic context, whereas 11.0% (117/1088) did not.

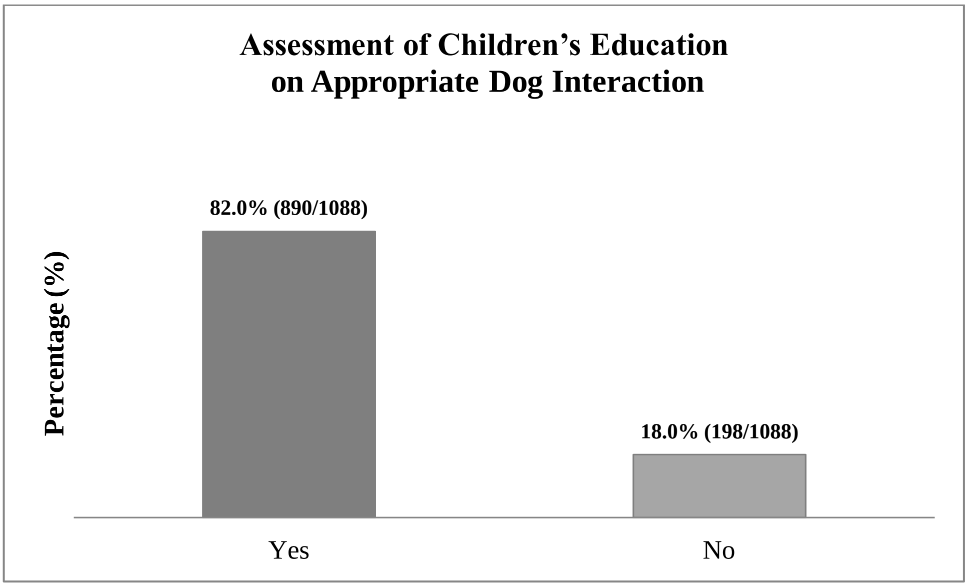

Figure S10. Assessment of children's education on appropriate dog interaction in the presence of children in the household. A total of 82.0% of respondents (890/1088) reported assessing whether children had been educated on appropriate interactions with dogs, whereas 18.0% (198/1088) did not.

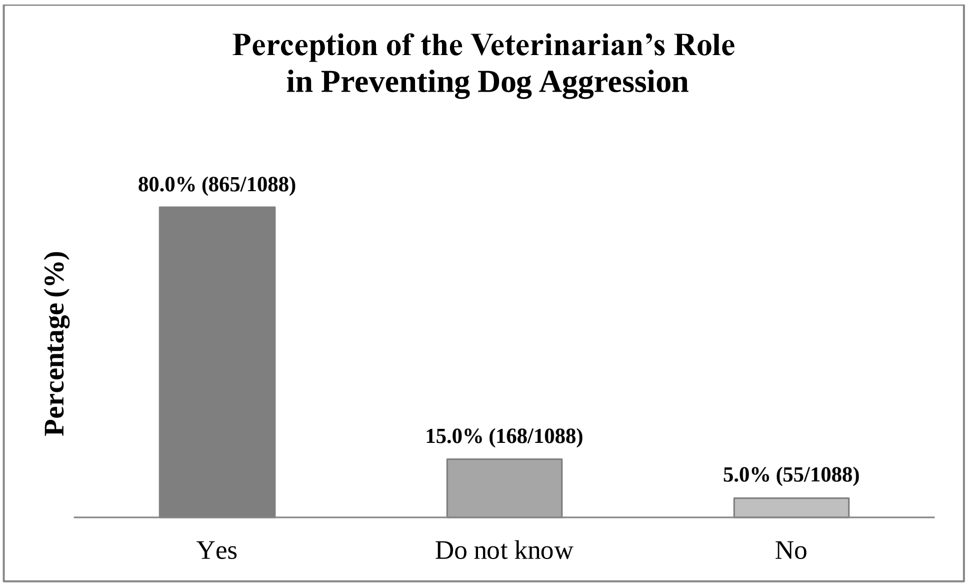

Figure S11. Perception of the veterinarian's role in preventing dog aggression. A total of 80.0% of respondents (865/1088) considered that the veterinarian should have an active role in the prevention of dog aggression, 15.0% (168/1088) reported uncertainty, whereas 5.0% (55/1088) did not consider this role necessary.

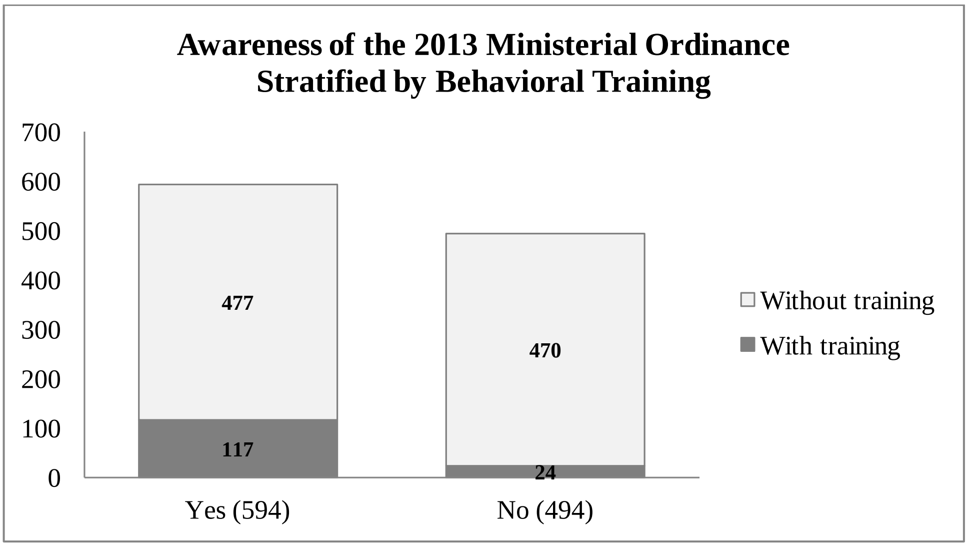

Figure S12. Awareness of the 2013 Ministerial Ordinance stratified by behavioral training. A total of 55.0% of respondents (594/1088) reported being familiar with the Ordinance, whereas 45.0% (494/1088) were not. Among those familiar with the Ordinance, 117 respondents had received training in behavioral medicine and 477 had not; among those not familiar, 24 had received training and 470 had not.

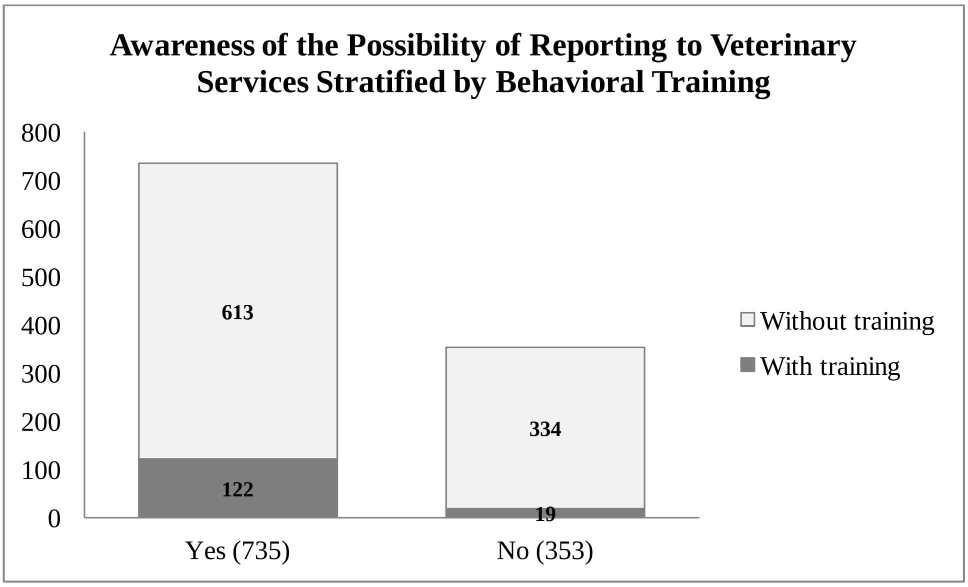

Figure S13. Awareness of the possibility of reporting to veterinary services stratified by behavioral training. A total of 68.0% of respondents (735/1088) reported being aware of the possibility of reporting to veterinary services, whereas 32.0% (353/1088) were not. Among those aware, 122 respondents had received training in behavioral medicine and 613 had not; among those not aware, 19 had received training and 334 had not.

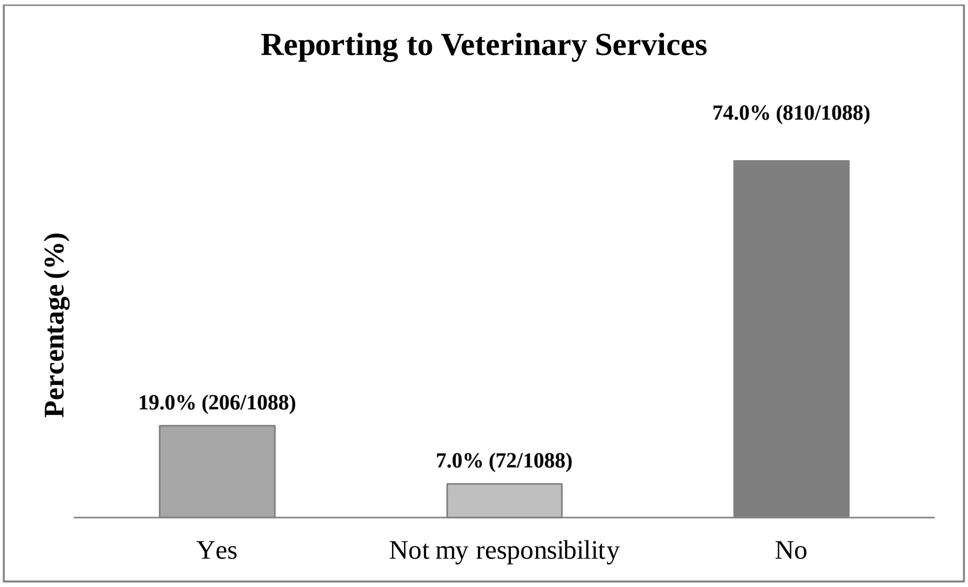

Figure S14. Reporting to veterinary services. A total of 19.0% of respondents (206/1088) reported having submitted at least one report to veterinary services, 7.0% (72/1088) considered reporting not to be their responsibility, whereas 74.0% (810/1088) had never submitted a report.

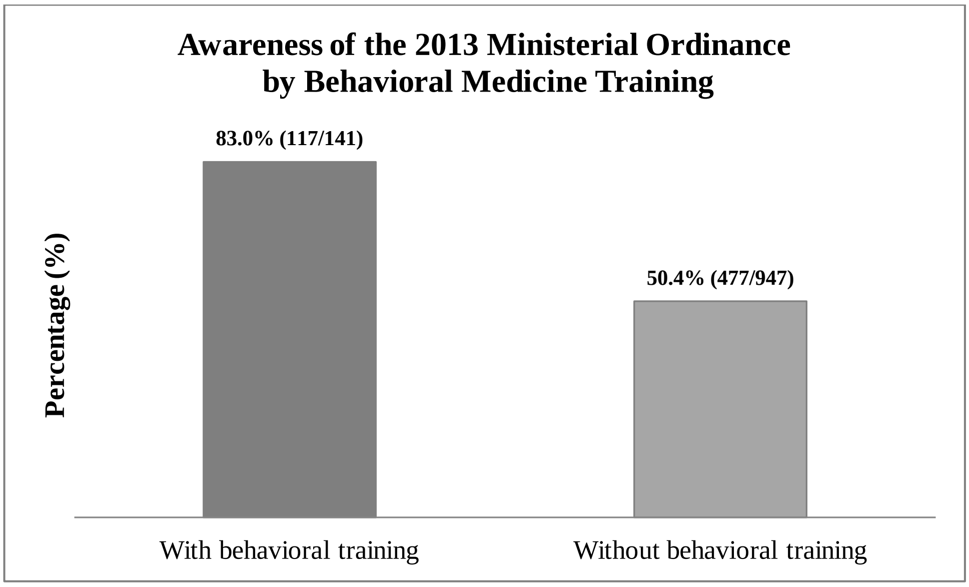

Figure S15. Awareness of the Italian Ministerial Ordinance of 6 August 2013 according to behavioral medicine training. Data are presented as percentages, with absolute numbers (n/N of respondents reporting knowledge within each group) in parentheses. The association between variables is statistically significant (χ^2 test, $p < 0.001$).

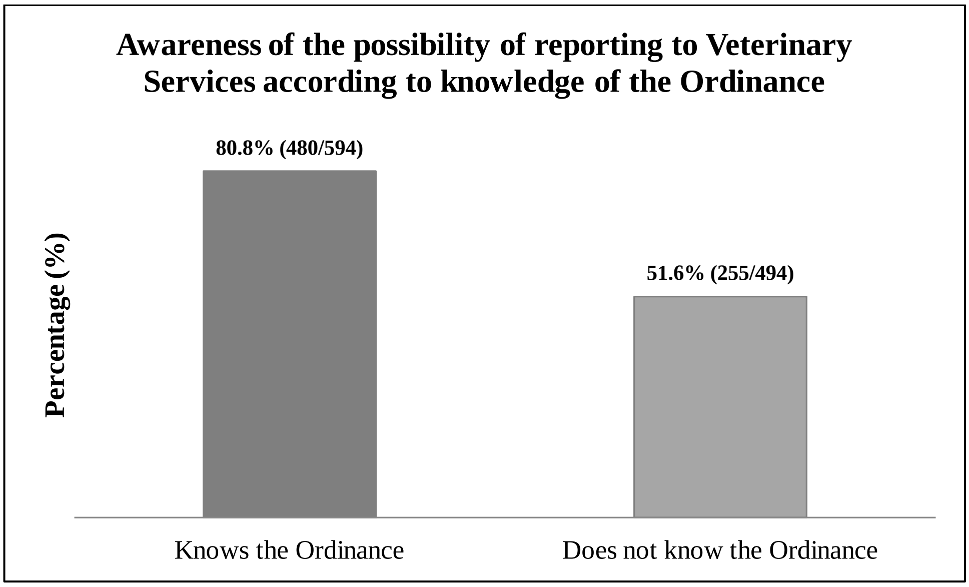

Figure S16. Awareness of reporting to Veterinary Services according to knowledge of the Italian Ministerial Ordinance of 6 August 2013. Data are presented as percentages, with absolute numbers (n/N of respondents reporting awareness within each group) in parentheses. The association between variables is statistically significant (χ^2 test, $p < 0.001$).

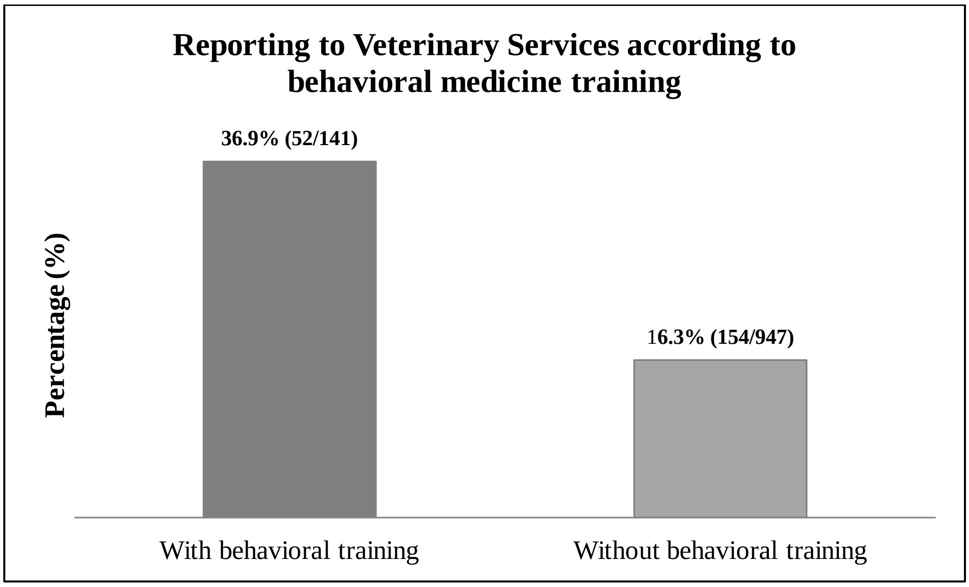

Figure S17. Having submitted at least one report to Veterinary Services according to behavioral medicine training. Data are presented as percentages, with absolute numbers (n/N of respondents reporting at least one report within each group) in parentheses. The association between variables is statistically significant (χ^2 test, $p < 0.001$).

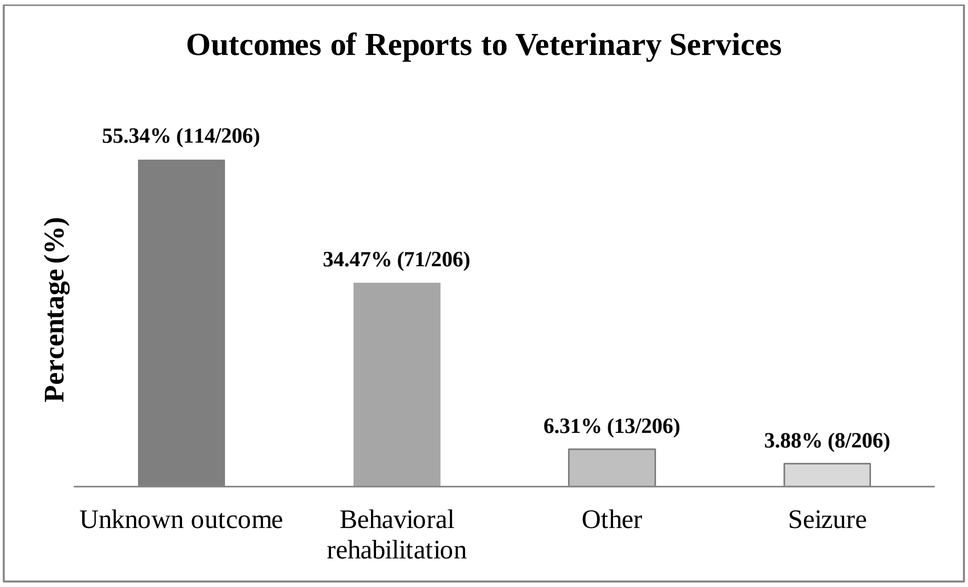

Figure S18. Outcomes of reports to veterinary services. Among the 206 respondents who had submitted at least one report, 55.34% (114/206) were not aware of the outcome, 34.47% (71/206) reported the implementation of a behavioral rehabilitation program, 6.31% (13/206) indicated other measures, and 3.88% (8/206) reported seizure of the animal.

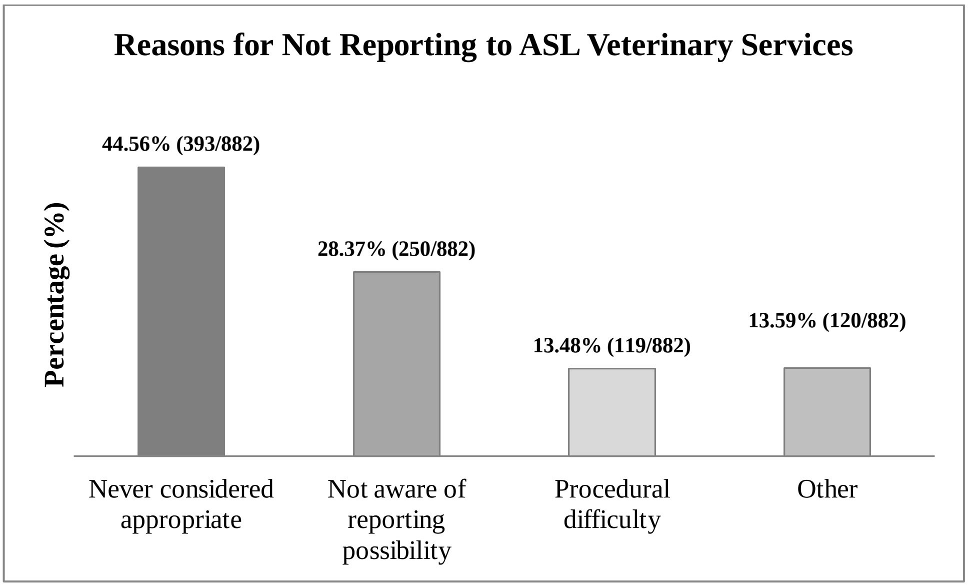

Figure S19. Reasons for not reporting to ASL veterinary services. Among the 882 respondents who had not submitted reports or considered it not within their responsibility, 44.56% (393/882) had never considered reporting appropriate, 28.37% (250/882) were not aware of the possibility of reporting, 13.48% (119/882) reported procedural difficulties, and 13.59% (120/882) indicated other reasons.

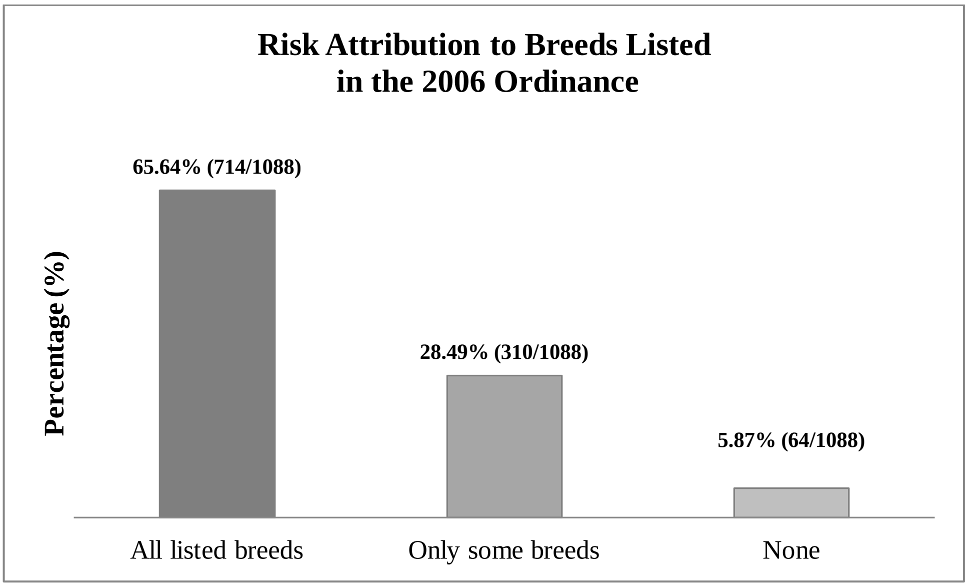

Figure S20. Risk attribution to breeds listed in the 2006 Ordinance. A total of 65.64% of respondents (714/1088) considered that all breeds included in the Ordinance may pose a risk if poorly managed, 28.49% (310/1088) indicated only some breeds, whereas 5.87% (64/1088) did not attribute a specific risk.

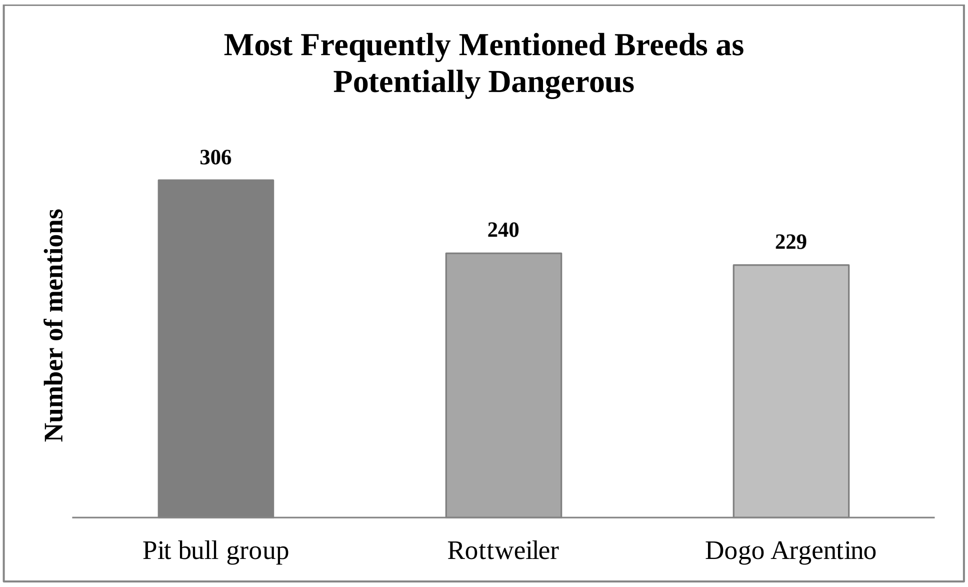

Figure S21. Most frequently mentioned breeds as potentially dangerous. Among the detailed responses, the most frequently reported breeds were the Pit bull group (306 mentions), followed by the Rottweiler (240) and the Dogo Argentino (229).

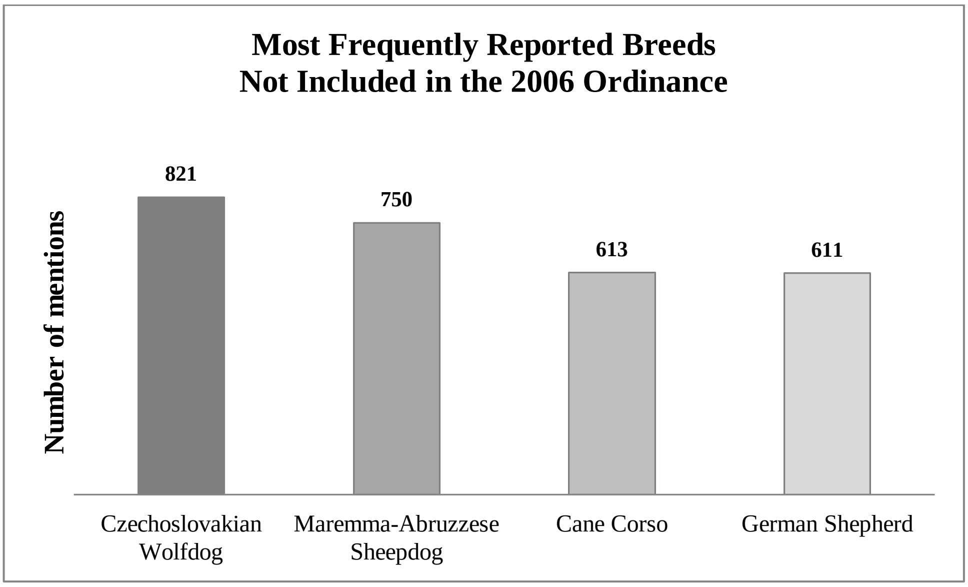

Figure S22. Most frequently reported breeds not included in the 2006 Ordinance. Among the detailed responses, the most frequently mentioned breeds were the Czechoslovakian Wolfdog (821 mentions), followed by the Maremma-Abruzzese Sheepdog (750), Cane Corso (613), and German Shepherd (611).

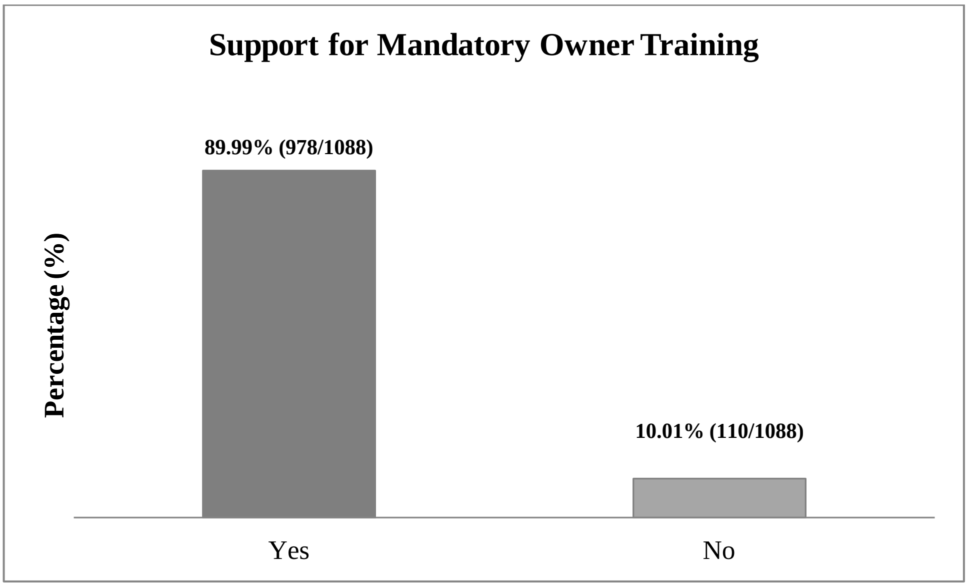

Figure S23. Support for mandatory owner training. A total of 89.99% of respondents (978/1088) were in favor of introducing mandatory training for dog owners, whereas 10.01% (110/1088) were opposed.

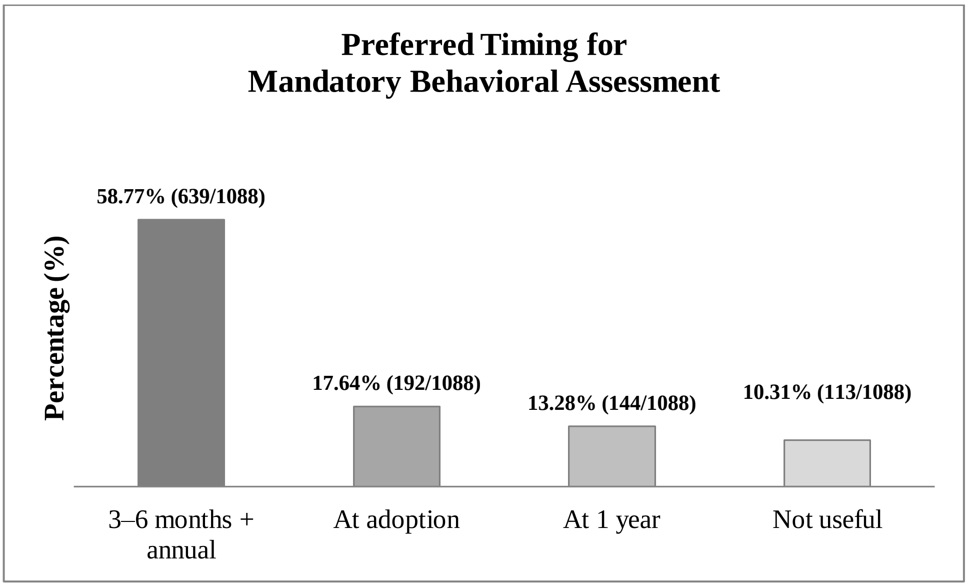

Figure S24. Preferred timing for mandatory behavioral assessment. A total of 58.77% of respondents (639/1088) indicated 3–6 months of age with annual follow-up as the preferred timing, 17.64% (192/1088) at the time of adoption, 13.28% (144/1088) at one year of age, whereas 10.31% (113/1088) did not consider this measure useful.

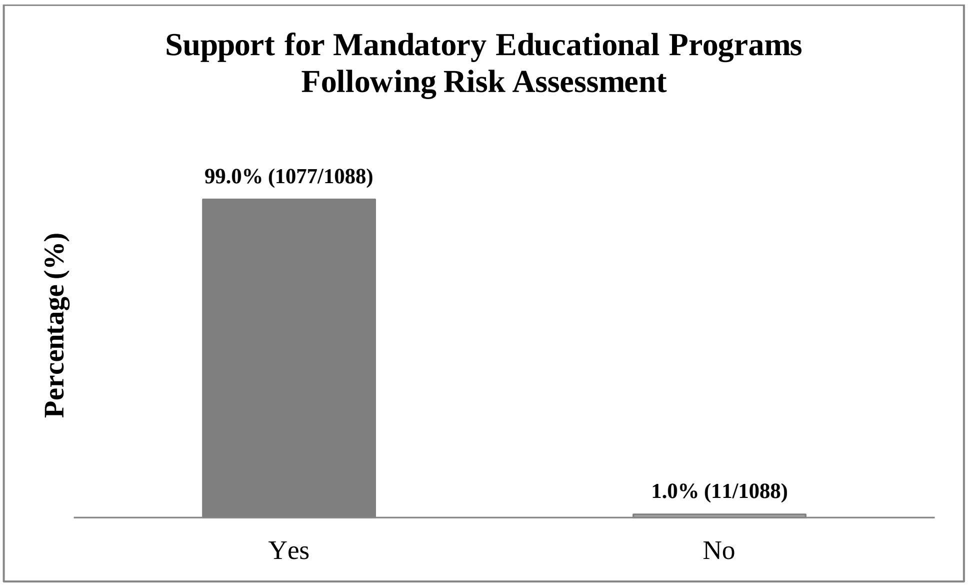

Figure S25. Support for mandatory educational programs following risk assessment. A total of 99.0% of respondents (1077/1088) were in favor of making educational programs mandatory in the presence of risk factors, whereas 1.0% (11/1088) were opposed.
